# The Cytochrome P450 OxyA from the Kistamicin Biosynthesis Cyclization Cascade is Highly Sensitive to Oxidative Damage

**DOI:** 10.3389/fchem.2022.868240

**Published:** 2022-04-08

**Authors:** Anja Greule, Thierry Izoré, Daniel Machell, Mathias H. Hansen, Melanie Schoppet, James J. De Voss, Louise K. Charkoudian, Ralf B. Schittenhelm, Jeffrey R. Harmer, Max J. Cryle

**Affiliations:** ^1^ Department of Biochemistry and Molecular Biology, The Monash Biomedicine Discovery Institute, Monash University, Clayton, VIC, Australia; ^2^ EMBL Australia, Monash University, Clayton, VIC, Australia; ^3^ ARC Centre of Excellence for Innovations in Peptide and Protein Science, Clayton, VIC, Australia; ^4^ Department of Chemistry, The University of Queensland, St Lucia, QLD, Australia; ^5^ Department of Chemistry, Haverford College, Haverford, PA, United States; ^6^ Monash Proteomics and Metabolomics Facility, Monash University, Clayton, VIC, Australia; ^7^ Centre for Advanced Imaging, The University of Queensland, St Lucia, QLD, Australia

**Keywords:** cytochrome P450, glycopeptide antibiotic biosynthesis, kistamicin, heme, biosynthesis

## Abstract

Cytochrome P450 enzymes (P450s) are a superfamily of monooxygenases that utilize a cysteine thiolate–ligated heme moiety to perform a wide range of demanding oxidative transformations. Given the oxidative power of the active intermediate formed within P450s during their active cycle, it is remarkable that these enzymes can avoid auto-oxidation and retain the axial cysteine ligand in the deprotonated—and thus highly acidic—thiolate form. While little is known about the process of heme incorporation during P450 folding, there is an overwhelming preference for one heme orientation within the P450 active site. Indeed, very few structures to date contain an alternate heme orientation, of which two are OxyA homologs from glycopeptide antibiotic (GPA) biosynthesis. Given the apparent preference for the unusual heme orientation shown by OxyA enzymes, we investigated the OxyA homolog from kistamicin biosynthesis (OxyA_kis_), which is an atypical GPA. We determined that OxyA_kis_ is highly sensitive to oxidative damage by peroxide, with both UV and EPR measurements showing rapid bleaching of the heme signal. We determined the structure of OxyA_kis_ and found a mixed population of heme orientations present in this enzyme. Our analysis further revealed the possible modification of the heme moiety, which was only present in samples where the alternate heme orientation was present in the protein. These results suggest that the typical heme orientation in cytochrome P450s can help prevent potential damage to the heme—and hence deactivation of the enzyme—during P450 catalysis. It also suggests that some P450 enzymes involved in GPA biosynthesis may be especially prone to oxidative damage due to the heme orientation found in their active sites.

## Introduction

**FIGURE 1:**
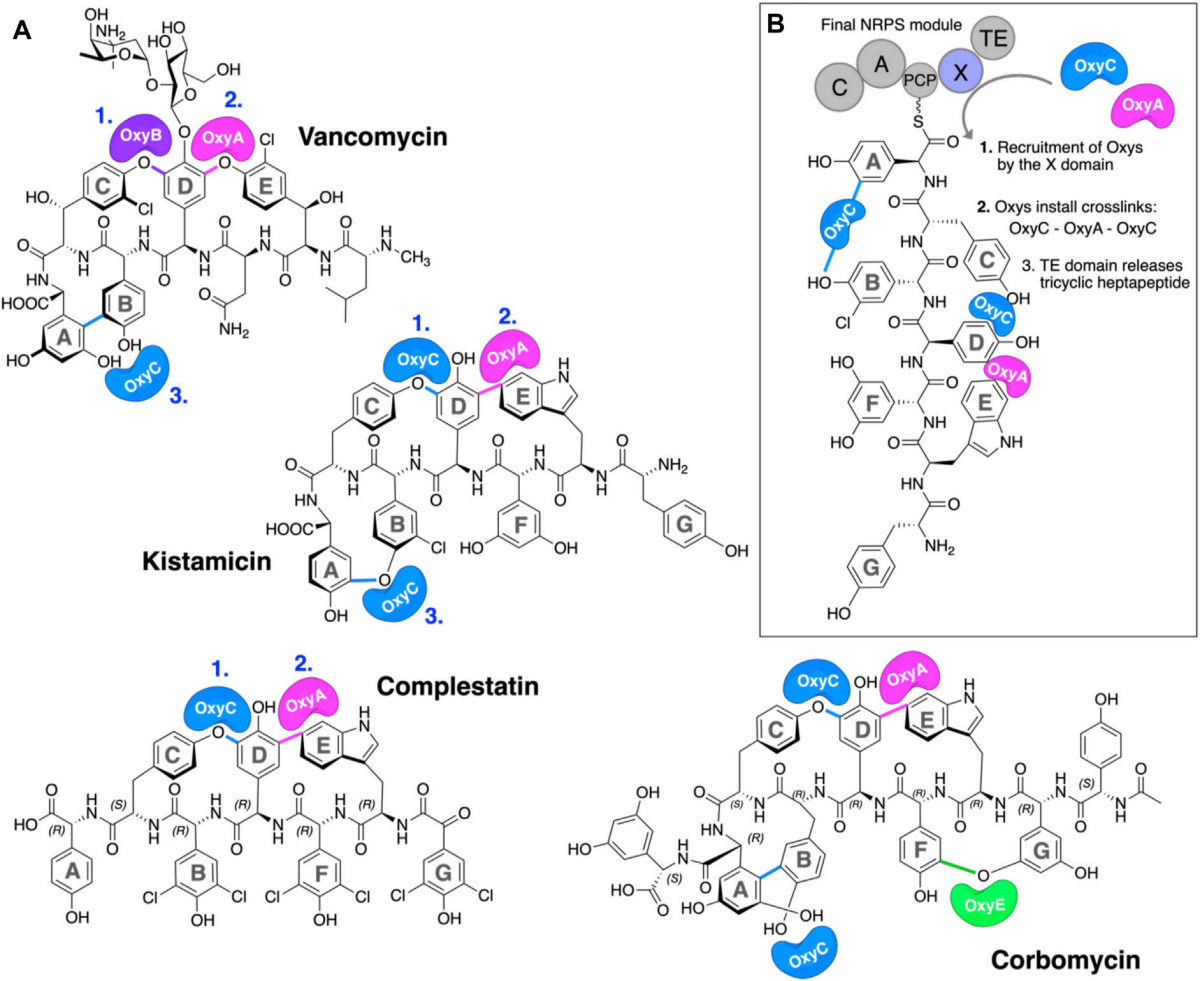
P450-mediated oxidative crosslinking during glycopeptide antibiotic biosynthesis. **(A)** Structure of vancomycin showing the P450-mediated oxidative crosslinking cascade—(OxyB: C–O–D, OxyA: D–O–E, and OxyC: AB) and Type V GPAs (i) kistamicin, whose biosynthesis exploits a bifunctional OxyC enzyme and an OxyA enzyme (OxyC: C–O–D, OxyA: DE, and OxyC: A–O–B); complestatin, whose biosynthesis exploits an OxyC enzyme and an OxyA enzyme (OxyC: C–O–D, and OxyA); and corbomycin, whose biosynthesis exploits a bifunctional OxyC enzyme, an OxyE enzyme, and an OxyA enzyme (tentatively OxyC: C–O–D, OxyE: F–O–G, OxyA: DE, and OxyC: A–O–B, exact timing has not been elucidated). The timing of the Oxy catalyzed crosslinks is indicated by blue numbers when known. [**(B)**, boxed] Side chain crosslinking reactions performed during glycopeptide antibiotic biosynthesis take place on the final non-ribosomal peptide synthetase (NRPS) module, while the heptapeptide is connected to a peptidyl carrier protein (PCP); this process requires the essential interaction of the Oxy enzymes with the X-domain (Oxy-recruitment domain), which is unique to GPA biosynthesis. Aromatic rings of the heptapeptide are labeled A–G. Abbreviations: A, adenylation domain; C, condensation domain; Oxy, cytochrome P450 enzyme; PCP, peptidyl carrier protein; TE, thioesterase domain; X, Oxy-recruitment domain.

The glycopeptide antibiotics (GPAs) are a complex class of non-ribosomal peptides with a range of antibiotic activity against gram-positive bacteria (Greule and Cryle, 2020). These (typically) heptapeptides contain a high proportion of amino acids with aromatic side chains and are particularly rich in non-proteinogenic phenylglycine (4-hydroxyphenylglycine (Hpg) and 3,5-dihydroxyphenylglycine (Dpg)) residues (Figure 1A) (Al Toma et al., 2015). In addition, these peptides also bear a number (2–4) of aryl and/or phenolic crosslinks between the side chains of aromatic residues, which serve to rigidify their structures and which are, in turn, essential for their antibiotic activity (Zhao et al., 2021). While most GPAs are known to inhibit bacterial cell wall division through complex formation with the D-Ala–D-Ala terminus of the cell wall precursor Lipid II (such as the clinically relevant compounds vancomycin and teicoplanin, known as type I–IV GPAs), a subgroup of GPAs containing the compounds complestatin (Chiu et al., 2001), kistamicin (Greule et al., 2019), and corbomycin (Culp et al., 2020) (known as type-V GPAs) display altered peptide structures that include a crosslinked tryptophan residue (Figure 1A). These GPAs have recently been demonstrated to exhibit a different type of antibiotic activity by inhibiting the activity of autolysins, peptidoglycan hydrolases responsible for cell wall remodeling during bacterial growth (Culp et al., 2020). Given this novel mode of activity, such GPAs are of great interest to investigate, both in terms of their structure/activity relationships and their biosynthesis.

Because of their important roles in medicine and as their commercial production remains through the fermentation of producer strains, GPA biosynthesis has been closely investigated (Greule and Cryle, 2020). At the heart of the biosynthesis is a non-ribosomal peptide synthetase (NRPS) assembly line that synthesizes the peptide backbone of GPAs in a stepwise manner (Süssmuth and Mainz, 2017). NRPS biosynthesis centers around groups of repeating catalytic domains—termed modules—that typically serve to introduce a single amino acid into the final peptide. Unlike ribosomal peptide synthesis, NRPS assembly lines directly encode the peptide sequence to be synthesized within the enzymatic domains of the machinery itself. Central to NRPS-mediated biosynthesis is the role of peptidyl carrier proteins (PCPs), which serve as the attachment point for all biosynthetic intermediates during peptide assembly (Izoré and Cryle, 2018). These intermediates are tethered to the PCP domains as thioesters *via* the 4-phosphopantethine (PPant) posttranslational modification found on all such carrier proteins (Süssmuth and Mainz, 2017). Generation of amino acyl–bound PCPs is performed by adenylation (A) domains in a two-step process, first commencing with the selection and activation of the specific amino acid monomer required at the specific stage of NRPS-mediated biosynthesis with consumption of ATP. Having formed a reactive mixed anhydride with AMP, the amino acid is then loaded onto the PCP *via* the attack of this reactive anhydride by the thiol terminus of the PPant arm of the neighboring PCP. From here, aminoacyl-bound PCPs can undergo further modifications *in trans* by a range of enzymes (including halogenases and hydroxylases) (Uhlmann et al., 2013; Kittilä et al., 2017; Kaniusaite et al., 2019) prior to their incorporation into the peptide, which is mediated by condensation (C) domains. In this step, the amino group of the downstream (acceptor) aminoacyl-PCP attacks the thioester of the upstream (donor) amino acid/peptide, leading to peptide bond formation with concomitant transfer of the upstream group onto the downstream aminoacyl-PCP. Such peptidyl–PCP substrates can themselves serve as substrates for additional domains, most commonly seen with epimerization (E) domains, which serve to alter the stereochemistry of the C-terminal residue of the PCP-bound peptide from the (L) to the (D) form.

Once peptide assembly is complete, peptide cleavage from the NRPS usually occurs next in a process that is mostly mediated by thioesterase (TE) domains and that can serve to introduce further modification into these peptides (e.g., through cyclization). In this regard, GPAs represent a unique divergence from this typical NRPS biosynthesis route, for it is at this point in their biosynthesis that the insertion of the side chain crosslinks within the peptide is performed (Figure 1B). (Peschke et al., 2017) This process is mediated by the activities of several cytochrome P450 (P450) monooxygenases—termed Oxys—which act upon the PCP-bound peptide in the terminal module of the NRPS (Zerbe et al., 2004; Haslinger et al., 2015; Peschke et al., 2016a; Peschke et al., 2016b).

**FIGURE 2:**
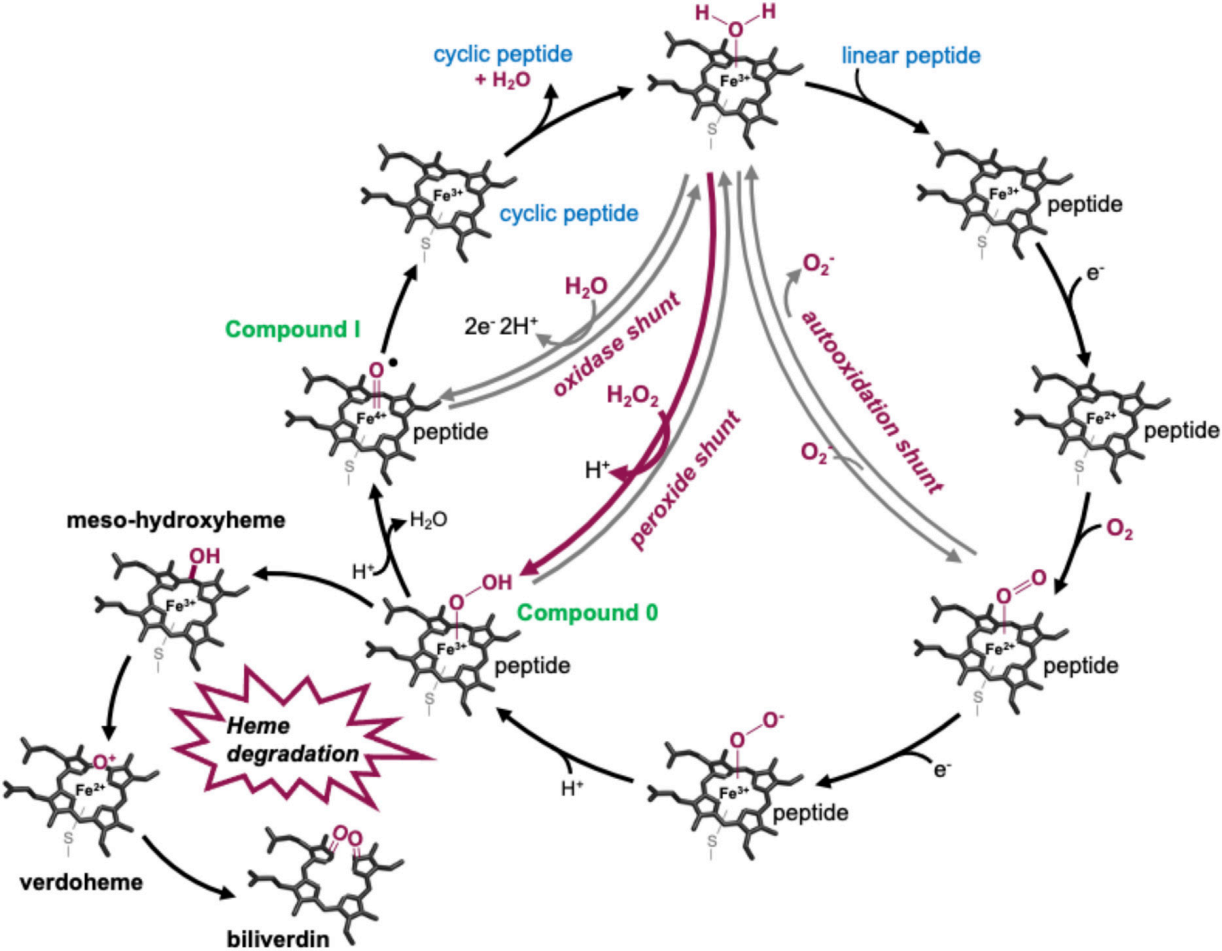
P450-active cycle exemplified for GPA crosslinking together with the heme oxygenase degradation pathway.

P450s are a superfamily of powerful oxidative hemoproteins that are widely distributed in nature and play diverse roles in biosynthetic processes in bacteria and fungi (Greule et al., 2018). Their prevalence in biosynthesis pathways stems from their unprecedented ability to regio- and stereo-selectively modify nonactivated C–H bonds in complex substrates. Their selectivity and specificity make them premiere biocatalysts, while their range of oxidative transformations is extensive—beyond the archetypal hydroxylation of C–H moieties. P450s are also known to catalyze epoxidation, heteroatom oxidation and aryl crosslinking (as seen in GPAs), among others (Greule et al., 2018). This reactive repertoire stems from their ability to generate a highly electrophilic oxidant—an iron (IV) oxo porphyrin cation radical, termed compound I—(Rittle and Green, 2010) through a complex, step-wise active cycle requiring molecular oxygen and two electrons, themselves delivered sequentially by redox partner enzymes (Figure 2). Within the P450 itself, the ligation of the heme iron—*via* a thiolate (i.e., deprotonated) cysteine side chain—is crucial for their ability to generate this extremely powerful oxidizing species (Green, 2009; Yosca et al., 2013), with modification of this cysteine side chain known to prevent the activity of such enzymes (Albertolle et al., 2017). Furthermore, P450s are carefully tuned to avoid autooxidation, especially given the number of Tyr residues found within these enzymes (Yosca et al., 2013). One ongoing area of research in understanding P450 mechanism is the characterization of the pathways of oxidation outside of the canonical C–H hydroxylation reaction, with epoxidation, sulfoxidation, and aromatic crosslinking showing the potential for alternate oxidation pathways. The mechanism through which the P450 (Oxy) enzymes perform the aromatic crosslinking in GPA biosynthesis has been investigated through several techniques (Geib et al., 2008; Holding and Spencer, 2008; Ali et al., 2020; Forneris et al., 2020), with the mechanism suggested to occur *via* two sequential 1-electron oxidation steps as opposed to a typical P450-mediated two-electron oxidation (Ali et al., 2020). The role of the readily abstractable phenolic/indolic protons appears highly important for this process (Forneris et al., 2020), while simultaneously raising the question of autooxidation of active site tyrosine residues in such P450 enzymes (Yosca et al., 2013).

Within GPA biosynthesis, P450s function in a specific, stepwise manner to install the essential side-chain crosslinks in the core peptide in a process that typically requires one Oxy enzyme per crosslink to be installed (Haslinger et al., 2015; Peschke et al., 2016a; Peschke et al., 2016b; Greule and Cryle, 2020). The recruitment of Oxy enzymes to the PCP-bound peptide substrate in these pathways is reliant on interactions of these P450s with the so-called X-domain, a C-type domain unique to the final module of GPA assembly lines (Haslinger et al., 2015; Greule et al., 2019). This interaction is required for the activity of almost all Oxy enzymes, and the discovery of this X/Oxy interaction was crucial to subsequent investigations that sought the reconstitution of GPA peptide crosslinking pathways *in vitro* (Zhao et al., 2021). While experiments studying the process of Oxy-mediated cyclization of peptide *in vitro* have provided a great deal of knowledge regarding the selectivity of this process for altered peptide sequences, challenges due to the complexity of the assay, hydrophobic nature of the peptides, and specific nature of the P450 catalysts have made these highly challenging to perform, in turn limiting the scale of the peptide products that can be isolated for further study (Forneris and Seyedsayamdost, 2018; Tailhades et al., 2020; Zhao et al., 2021). In this regard, one glaring omission from *in vitro* experiments into the GPA cyclization cascade is the successful incorporation of the 4-Hpg/Trp crosslink found in Type V GPAs. This limits both the ability to study the selectivity and mechanism of the OxyA enzymes responsible and further the access to homologs of these interesting GPAs *via* biomimetic synthesis. A previous study of the peptide crosslinking cascade in the Type V GPA kistamicin, while revealing important details around the conservation of the Oxy/X interface and dual cyclization activity of the OxyC enzyme in this system, was still unable to reconstitute OxyA activity despite showing good levels of activity for OxyC (Greule et al., 2019). Given that apparent oxidative damage occurs to the Oxy enzymes during *in vitro* reconstitution experiments (and can be averted in some cases through the inclusion of small molecules to reverse this damage) (Tailhades et al., 2019), we undertook here to assess the susceptibility of OxyA enzymes from Type V GPA biosynthesis to oxidative damage. Our results show that these types of OxyA enzymes are highly prone to oxidative damage by peroxide (a product of non-productive P450 activation) and that the incorporation of the heme moiety in these P450 enzymes appears connected to the presence of such damage. Furthermore, we show that OxyA activity can be reconstituted against small PCP-bound peptide substrates, indicating that the biomimetic synthesis of type V GPAs could well be in reach, provided these reactions are carefully controlled to avoid the generation of unwanted oxidizing species.

## Experimental

Site-directed mutagenesis of OxyA_kis*.*_ To generate the Tyr_99_ to Phe mutant of OxyA_kis_, the plasmid pET28a-OxyA_kis_ (6,461 bp) was amplified by PCR using the primer pair ATG​GTT​GCT​CCA​GCT​TtC​TCC​GTT​CGC​CGG​ATG​C (forward) and TTG​CAT​CCG​GCG​AAC​GGA​GaA​AGC​TGG​AGC​AAC​C (reverse). The methylated template DNA was digested by *DpnI* restriction endonuclease (NEB), and the linear PCR product was used to transform chemically competent *E. coli* NEB alpha cells. Positive clones were selected on kanamycin plates, and the desired mutation was verified by DNA sequencing of the isolated, purified plasmid.

Expression and purification of proteins. *P450 enzymes.* The cloning, expression, and purification of OxyA_kis_, Y99F OxyA_kis,_ and OxyC_kis_ were described previously (Greule et al., 2019). In brief, for all proteins, 10 L ZYM-50524 autoinduction media was inoculated with 1% (v/v) *E. coli* Arctic Express preculture, supplemented with 50 mg L^−1^ kanamycin and 0.1 g L^−1^ of the heme-precursor δ-aminolevulinic acid and incubated for 6 h at 37°C before reducing the temperature to 16°C and allowing the culture to incubate for a further 72 h. The cells were expressed at 120 rpm or 80 rpm shaking. After cell disruption *via* sonication, the P450 enzymes were purified by a combination of Ni-NTA affinity, anion ion exchange, and size exclusion chromatography using an ÄKTA purifier system (GE Healthcare) before being flash-cooled in liquid nitrogen and stored at −80°C.

*PCP-X construct.* The PCP-X construct from the kistamicin NRPS was expressed and purified as previously reported (Greule et al., 2019).

Verification of OxyA_kis_ Y99F mutation by protein mass spectrometry. OxyA_kis_ Y99F was subjected to a tryptic digest and peptide fragments analyzed by a nano LC system (Agilent 1200 series nano) using a Zorbax 300SB-C18 (75 μm × 15 cm, nanoViper, C18, 3 μm, Agilent Technologies) with a trap column Acclaim PepMap 100 (100 μm × 2 cm, nanoViper, C18, 5 μm, 100 Å; Thermo Scientific). The protein masses were detected on a mass spectrometer MicroTOFq (Bruker Daltonics), and peptide fragments were analyzed by MASCOT V2.4 (Matrix Science); SI Supplementary Figure S4.

Spectroscopic analysis of P450 enzymes after reduction and CO complexation. Reduced, CO-bound spectra of Y99F OxyA_kis_ were obtained using a Jasco V-750 spectrophotometer at 30°C. The enzymes were diluted to 2.5 µM in Tris-HCl (50 mM, pH 7.4), reduced using 10 µL of a saturated solution of sodium dithionate (Sigma) and CO generated by the addition of a small quantity of solid sodium boranocarbonate (Dalton Pharma Services). The UV/Vis spectra were then measured between 390 and 900 nm.

Assessing peroxide-mediated damage of P450 enzymes. Oxy enzymes were diluted to 1 µM (data shown in Figures 6, 7) or 4 µM (data shown in Figure 7) in 50 mM Tris-HCl (pH 7.4) in a final volume of 500 μL. Different concentrations of H_2_O_2_ (0.4–40 mM) or a saturated *m*-CPBA solution were then added, and the UV/Vis spectra were measured between 390 and 900 nm after various time points using a Jasco V-750 spectrophotometer at 30°C.

**FIGURE 3:**
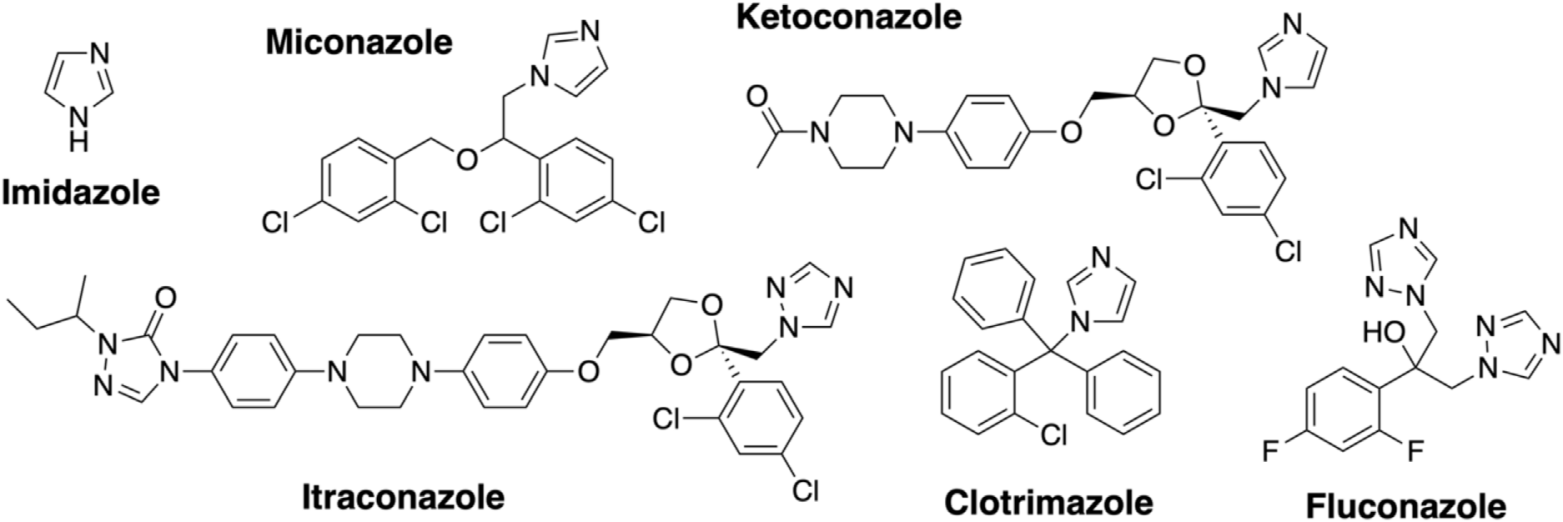
Azole inhibitors used in this study.

Azole inhibitor binding to the Oxy enzymes using UV–Visible spectroscopy. Imidazole (Sigma) was dissolved in water (10 mM stock). Clotrimazole, ketoconazole, fluconazole, miconazole, and itraconazole (Abblis Chemicals LLC, Figure 3) were freshly dissolved in DMSO (0.1–10 mM stock solutions). OxyA_kis_ and OxyC_kis_ were diluted to 2.5 µM in 2 mL Tris-HCl (50 mM) at pH 8 and split into two cuvettes. The spectra were obtained using a dual Jasco V-750 spectrophotometer at 30°C. Different concentrations of the azole compound were added to one cuvette, while in the second cuvette, the same volume of DMSO was added to the protein solution. The spectra were measured between 350 and 600 nm after an equilibration period of 2 min. The absorbance difference ∆A between A_max_ and A_min_ was extrapolated and plotted against the azole concentration (∆A = A_max_−A_min_); see SI Supplementary Figure S1. The maximal amplitudes (∆A_max_) and dissociation constants (K_d_) were determined as reported for OxyB_tei_ and OxyA_tei_.

Electron paramagnetic resonance (EPR) spectroscopy. CW EPR experiments were carried out on a Bruker Elexsys E500 spectrometer equipped with an ElexSys Super High Sensitivity Probehead and an LHe Oxford Instruments cryostat. The magnetic field was calibrated with 2,2-diphenyl-1-picrylhydrazyl (*g* = 2.0036), and measurements were carried out at 7.5 K using a modulation amplitude of 0.5 mT, modulation frequency of 100 kHz, and nonsaturating microwave power of 2 mW (20 dB of 200 W). Simulation of the low-spin P450 signals was carried out with the XShophe (Hanson et al., 2004). The enzyme (200 μL/200 μM in an Eppendorf tube) was treated with 20 μL of 0.3% H_2_O_2_ and allowed to react for a specified time before 50 μL of the solution was transferred to a quartz EPR tube and flash-frozen in liquid N_2_ for CW EPR measurements.

Crystallization, data collection, and structure determination of OxyA_kis_. The OxyA protein in Tris-HCl (50 mM, pH 7.8) NaCl (200 mM) (15 mg/ml) was crystallized using a sitting-drop vapor diffusion method. Initial screening was performed at the Monash Molecular Crystallization Facility (MMCF), with subsequent optimization performed manually in 48-well sitting-drop plates. The complex was crystallized using a sitting-drop vapor diffusion method by mixing 1 µL protein with 1 µL of an optimized reservoir solution containing 0.1 M MMT buffer (molar ratios 1:2:2—DL-malic acid: MES: Tris base pH 6) and 25% PEG 1500. Red crystals formed after 1 week at 20°C. The crystals were cryoprotected by transfer in a drop made of the reservoir solution supplemented with glycerol (30% final concentration, *v/v*). The crystals were collected in cryoloops and flash-frozen in liquid nitrogen. Data were collected at the Australian Synchrotron (Clayton, Victoria, Australia) on beamline MX1 at 100K. Data processing was performed using XDS (Kabsch, 2010) and AIMLESS as implemented in CCP4 (Collaborative Computational Project Number 4, 1994). The phases were obtained in a molecular replacement experiment using a PHENIX in-built Phaser module (Adams et al., 2010) and with a model generated by PHYRE (Kelley et al., 2015). The structure was built and refined using COOT (Emsley and Cowtan, 2004) for model building and PHENIX-refine for refinement. All graphics were generated using Pymol (Schrödinger LLC). The sequence alignments were generated using Clustal Omega (Sievers et al., 2011). Data are shown in SI Supplementary Table S1.

Soaking of OxyA_kis_ crystals with imidazole. Imidazole was freshly dissolved in DMSO with a final concentration of 10 mM and further diluted to 2 mM in the mother liquor condition + glycerol. OxyA_kis_ crystals were prepared as previously indicated and soaked/cryoprotected in the solution containing the inhibitor. Data collection was performed as described previously.

**FIGURE 4:**
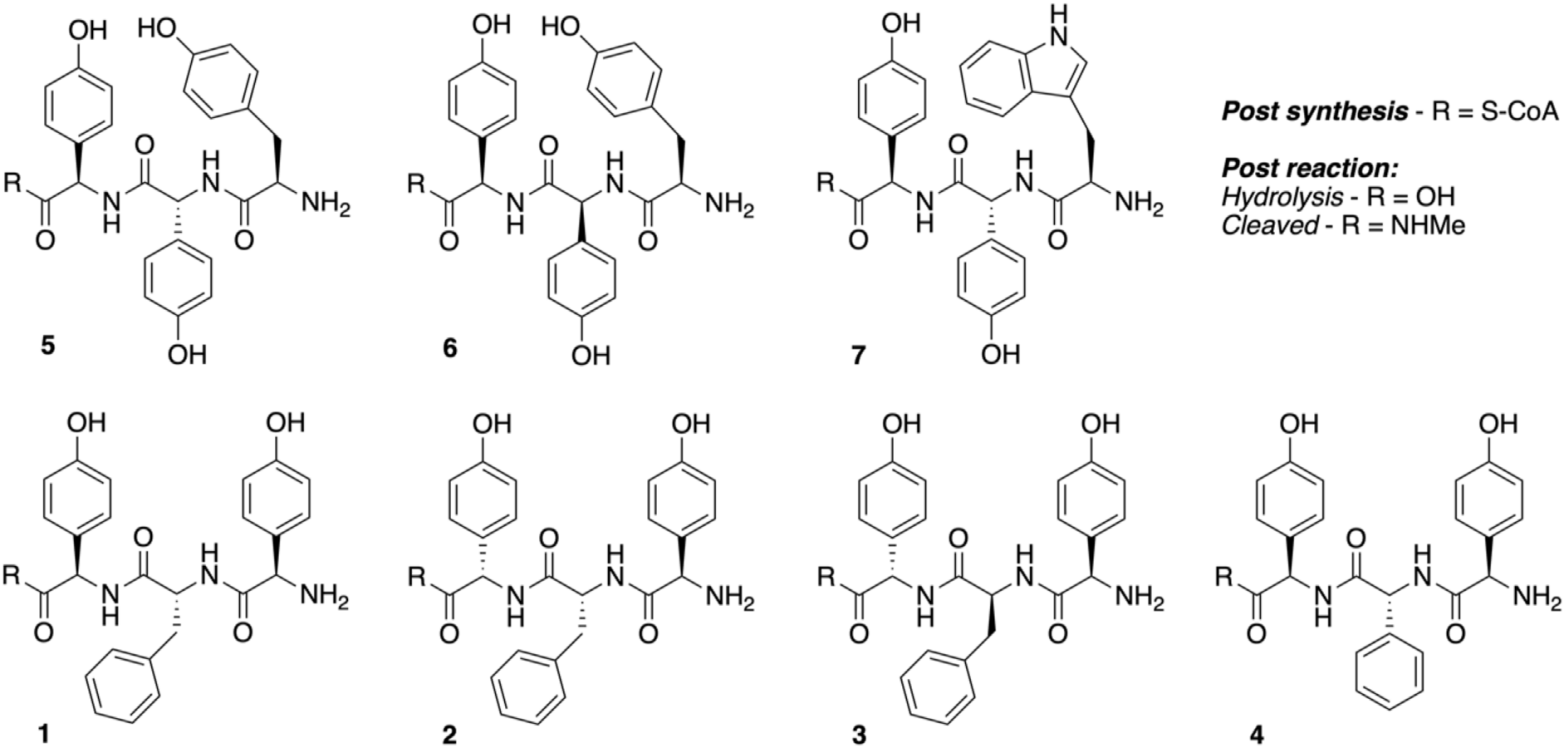
Structures of tripeptides 1–7 synthesized in this study.

Peptidyl synthesis and turnover. Seven tripeptide CoA thioesters (**1−7**, **R = S-CoA**; Figure 4) were synthesized using the previously reported method for phenylglycine-containing peptides (Brieke and Cryle, 2014; Tailhades et al., 2018) before being enzymatically loaded by the phosphopantetheinyl transferase Sfp (R4-4 mutant) (Sunbul et al., 2009) onto the carrier protein domain of a PCP-X di-domain from the kistamicin NRPS. Turnover of these peptidyl-PCP-X substrates by both OxyA_kis_ and OxyC_kis_ was performed as previously reported (Greule et al., 2019), with the thioester-tethered peptide products liberated as methylamide species (R = NHMe) *via* the addition of methylamine prior to solid-phase extraction and HRMS analysis.

High-resolution mass spectrometry (HRMS) analysis. All high-resolution mass spectrometry measurements were performed on a QExactive Plus mass spectrometer (Thermo Scientific) coupled to a Dionex UltiMate 3,000 RSLCnano system equipped with a Dionex UltiMate 3000 RS autosampler, an Acclaim PepMap RSLC analytical column (75 μm × 50 cm, nanoViper, C18, 2 μm, 100 Å; Thermo Scientific), and an Acclaim PepMap 100 trap column (100 μm × 2 cm, nanoViper, C18, 5 μm, 100 Å; Thermo Scientific). The samples were separated by increasing concentrations of 80% acetonitrile/0.1% formic acid at a flow of 250 nL/min over 30 min. The instrument was operated in alternating data-dependent acquisition (DDA) and parallel reaction monitoring (PRM) cycles, such that for each ms1 precursor scan, five ms2 scans preceded several targeted PRM scans to ensure fragmentation of predefined, sample-dependent m/z precursors. Each survey ms1 scan (250–1,200 m/z) was acquired with a resolution of 70,000 and a normalized AGC (automatic gain control) target of 1e6. Dynamic exclusion was set to 10 s after one occurrence. The five most intense ions were selected for HCD fragmentation (fixed collision energy mode, 24 HCD collision energy) with a resolution of 17,500 and a normalized AGC target of 1e5. Subsequent targeted PRM scans were acquired with essentially identical settings. The raw data files were analyzed with QualBrowser (XCalibur 3.0.63, Thermo Scientific) to view the spectra and generate extracted ion chromatograms.

## Results and Discussion

### Assessing the Heme Environment of the Kistamicin Oxy Enzymes

**FIGURE 5:**
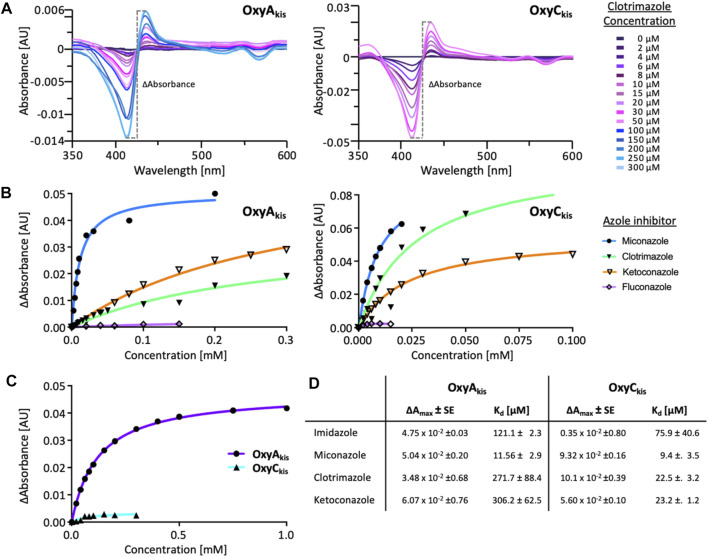
UV–Visible spectroscopic binding studies of OxyA_kis_ and OxyC_kis_ with azole inhibitors. **(A)** Example for the spectral response of OxyA_kis_ (left) and OxyC_kis_ (right) upon different concentrations of clotrimazole. **(B)** Amplitudes of the binding of various azole inhibitors to OxyA_kis_ (left), OxyC_kis_ (right), and imidazole **(C)**. Inhibitors tested: imidazole, miconazole, clotrimazole, ketoconazole, fluconazole, and itraconazole. **(D)** Summary of azole binding to OxyA_kis_ and OxyC_kis_. ∆A_max_ and K_D_ derived from the one-site binding model. SE, standard error of the regression.

Given that we had observed a significant difference in activity between the OxyC and OxyA enzymes from kistamicin biosynthesis (OxyC_kis_ and OxyA_kis_), we first undertook an analysis of the Oxy active sites using the binding of various azole inhibitors to probe the accessibility of the heme iron for these different structures (Figures 3, 5; Supplementary Figure S1). For the small inhibitor imidazole, OxyA_kis_ showed significant direct binding to the heme iron that was not observed for OxyC_kis_, which resulted in an order of magnitude increase in the different absorption spectra observed with OxyA_kis_, despite the affinity being lower for OxyA_kis_ (OxyA_kis_ K_D_ = 120 μM vs. OxyC_kis_ = 84 μM). In general, OxyC_kis_ displayed higher affinity to all inhibitors than OxyA_kis_, although these values were lower for both enzymes than those reported for the comparable teicoplanin enzymes OxyA_tei_ and OxyB_tei_ (Haslinger et al., 2014; Haslinger and Cryle, 2016). These data indicate that the heme environment of OxyA_kis_ is significantly different compared to other Oxy enzymes analyzed to date.

### OxyA_kis_ is Highly Sensitive to Hydrogen Peroxide

**FIGURE 6:**
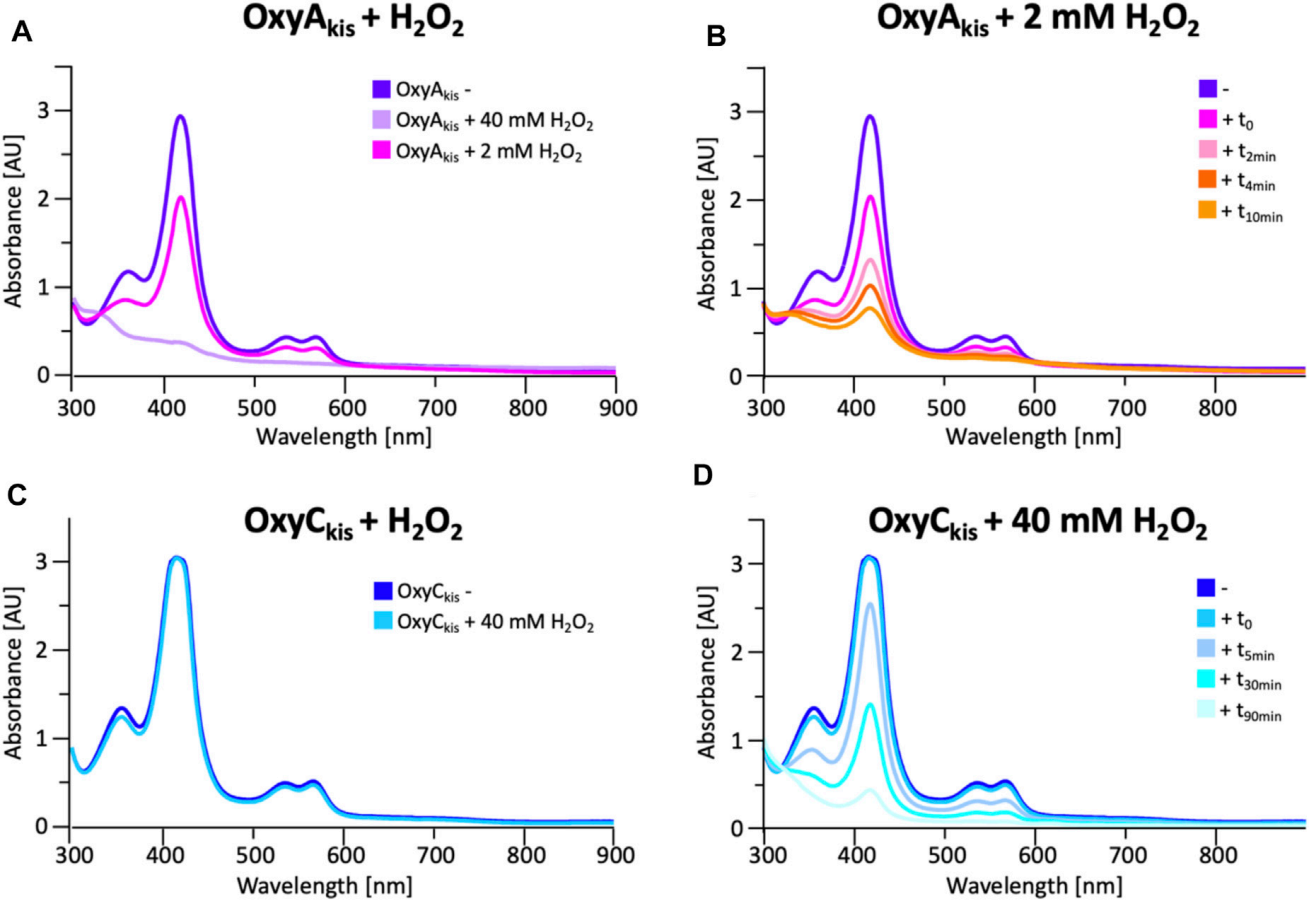
Initial assessment of the effect of hydrogen peroxide addition to the UV/Visible spectra of OxyA_kis_ and OxyC_kis_. **(A)** UV/Vis spectra of OxyA_kis_ after addition of varying amounts of hydrogen peroxide (t_0_). UV/Visible spectra shown from 300 to 900 nm **(B)** Effect over time of addition of 2 mM hydrogen peroxide on the UV/Vis spectra of OxyA_kis_ (300–900 nm). **(C)** UV/Vis spectra of OxyC_kis_ after addition of 40 mM hydrogen peroxide (t_0_). UV/Visible spectra shown from 300 to 900 nm **(D)** Effect over time of addition of 40 mM hydrogen peroxide (20x that used with OxyA_kis_) on the UV/Vis spectra of OxyC_kis_ (300–900 nm).

To date, OxyA_kis_ activity had not been observed *in vitro* despite the enzyme showing the requisite 450-nm spectrum upon reduction and CO complex formation, which indicates that the enzyme is expressed in a catalytically competent form. Given that the reconstitution system used with such Oxy enzymes is not natural to the kistamicin producer, we were curious whether this could be causing enzyme inactivation through oxidative damage. Indeed, recent optimization of the GPA cyclization cascade *in vitro* has shown the importance of protecting these P450 enzymes from inactivation over long reactions, presumably caused by oxidative damage (Tailhades et al., 2019). To analyze the sensitivity of both OxyA_kis_ and OxyC_kis_ (that has been shown to be catalytically competent), hydrogen peroxide (H_2_O_2_) was titrated into solutions of both enzymes, and the resultant damage of the proteins was monitored by a decrease in the absorbance of the 418-nm heme Soret absorbance peak by UV/Vis absorbance spectroscopy (Figure 6).

**FIGURE 7:**
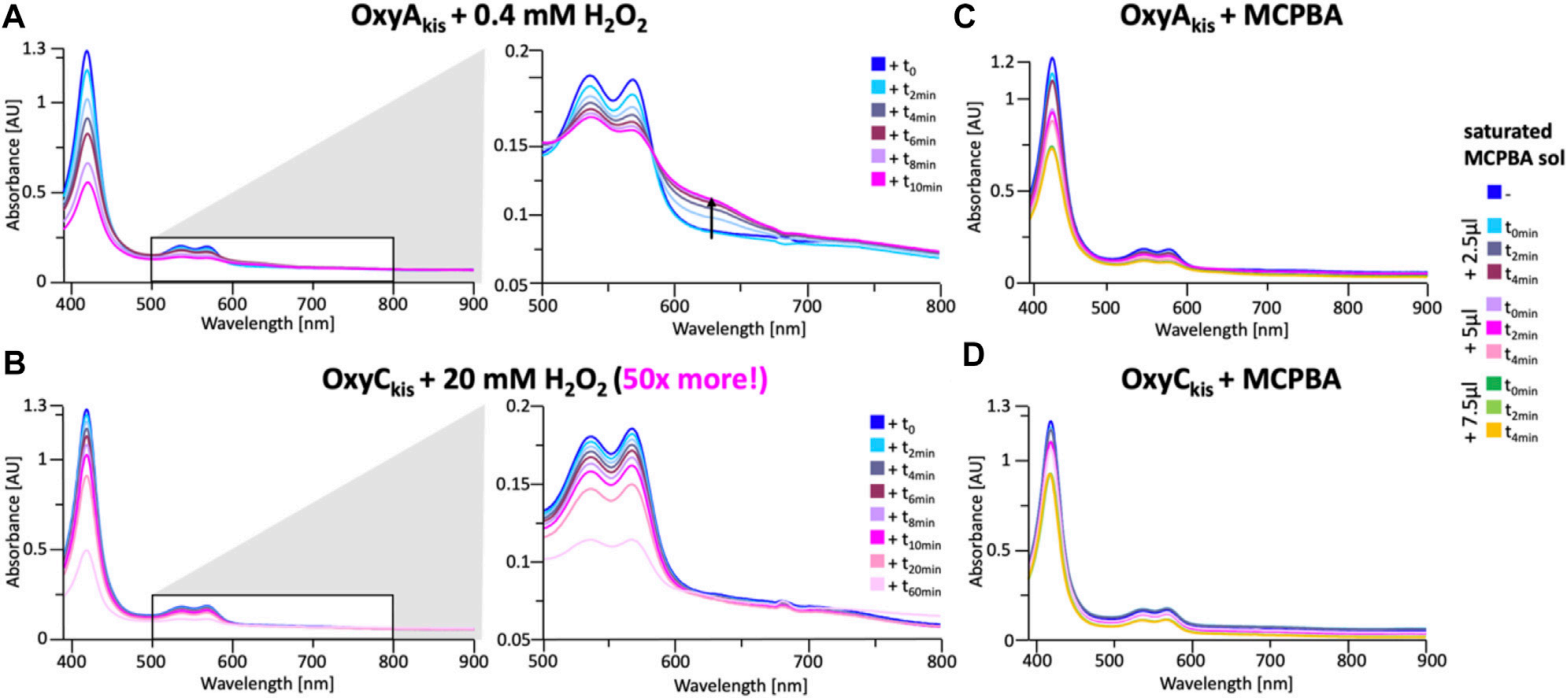
Time-dependent UV/Visible spectra of OxyA_kis_ and OxyC_kis_ after addition of hydrogen peroxide and *m*-CPBA. **(A)** UV/Vis spectra of OxyA_kis_ after addition of 0.4 mM hydrogen peroxide at different time points. UV/Visible spectra shown from 390 to 900 nm (left panel) and a zoomed view of 500–800 nm (center panel). The shoulder in absorbance between 600 and 700 nm is indicated by an arrow. **(B)** UV/Vis spectra of OxyC_kis_ after addition of 20 mM hydrogen peroxide (50 times more than OxyA_kis_) at different time points. UV/visible spectra shown from 390 to 900 nm (left panel) and a zoomed view of 500–800 nm (center panel). It is to be noted that no shoulder in the absorbance signal is present. UV/Vis spectra of **(C)** OxyA_kis_ and **(D)** OxyC_kis_ after addition of a saturated m-CPBA solution.

**FIGURE 8:**
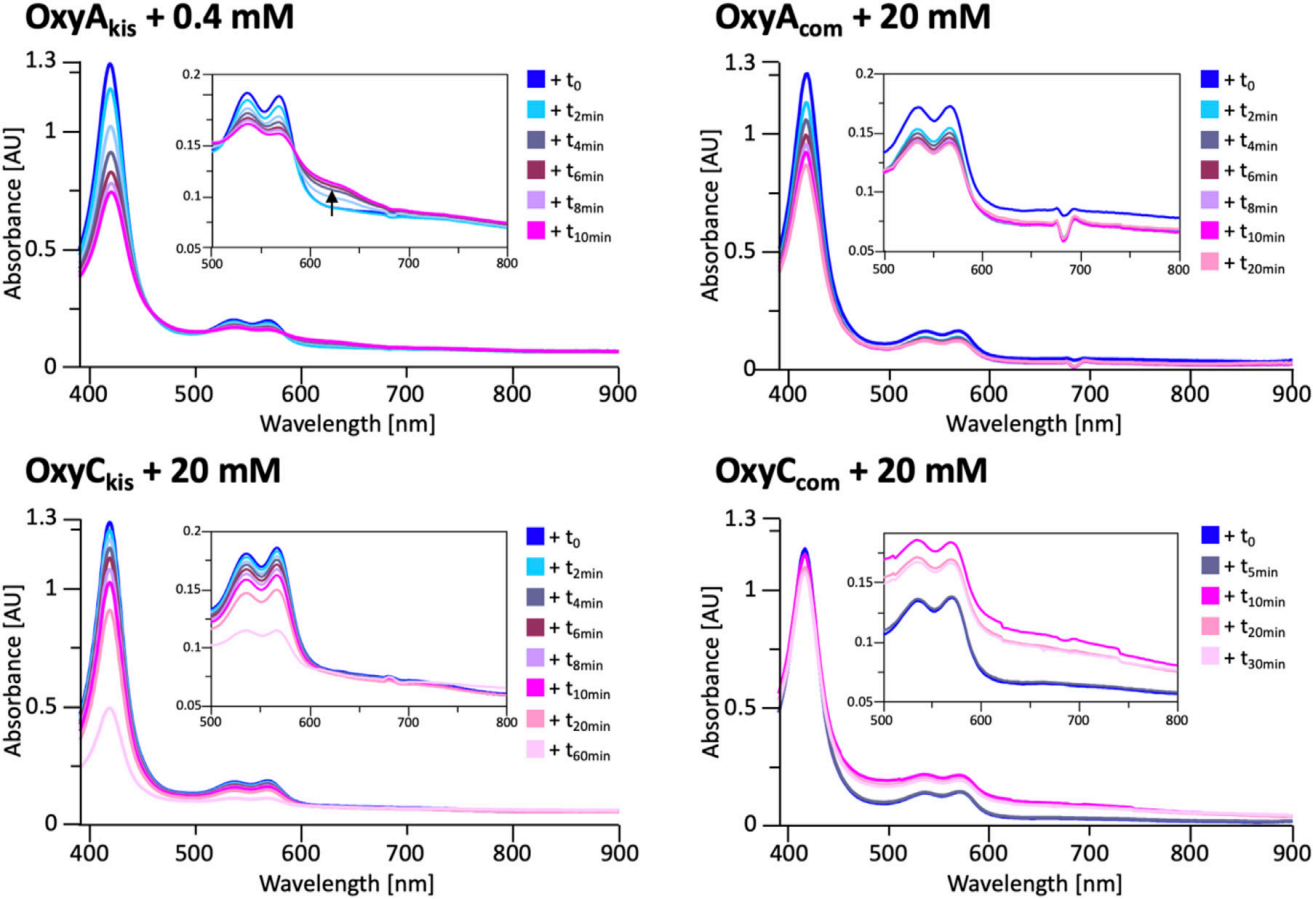
UV/Vis spectra measurement of OxyA_kis_ and OxyC_kis_ and the complestatin homologs OxyA_com_ and OxyC_com_ after addition of hydrogen peroxide. The inset in each panel shows the absorption region between 500 and 800 nm, revealing that the unusual shoulder between 600 and 700 nm is only observed in the case of OxyA_kis_.

These experiments showed that there was an obvious difference between these two P450 enzymes. While the addition of a solution of 40 mM hydrogen peroxide led to no obvious change within OxyC_kis_, the heme signal in the OxyA_kis_ sample is lost almost instantaneously (Figure 6). The use of solutions with a much lower concentration of hydrogen peroxide still led to rapid bleaching of the heme absorption in OxyA_kis_, with almost no absorbance observed after 10 min (Figure 7A). OxyC_kis_ is clearly much more stable than OxyA_kis_ to oxidative reagents, as even the use of 20-fold more hydrogen peroxide leads to slower heme bleaching (Figure 7B). The addition of *m*-CPBA does not have such a drastic effect on the Oxy enzymes, with both proteins showing comparable decrease of absorbance (Figures 7C, D). This degradation also displays a different trend to that observed with hydrogen peroxide for the immediate reduction in absorbance upon *m*-CPBA addition does not continue over time. A closer investigation of the UV/Vis spectra of the P450s after hydrogen peroxide treatment also reveals differences between OxyA_kis_ and OxyC_kis_ in the absorption range between 500 and 700 nm. While the β/α bands at 539 and 566 nm of the H_2_O_2_-treated samples decline in the spectra of both enzymes upon peroxide addition, the spectrum of OxyA_kis_ shows an increase in absorbance between 600 and 700 nm (Figure 8, indicated by an arrow), that is not present in OxyC_kis_. Such an increase is reminiscent of the absorption spectra of the verdoheme intermediate formed by the heme-degrading enzyme heme oxygenase, and although the absorption shoulder present in the OxyA_kis_ spectra is not as significant as in the case of heme oxygenase, this could be due to the instability of such heme species under aerobic conditions (Matsui et al., 2005; Uchida et al., 2017). It is curious that this absorbance shoulder is not present in the *m*-CPBA–treated solutions.

To determine whether these responses to peroxide treatment are general for these types of P450s from the biosynthesis of type-V GPAs, the complestatin Oxy homologs ComI (OxyA_com_) and ComJ (OxyC_com_) were also treated with hydrogen peroxide and their spectra analyzed (Figure 8; SI Supplementary Figure S2 and Supplementary Figure S3). (Chiu et al., 2001; Mollo et al., 2017) Measurement of OxyC_com_ was challenging because of gas evolution in the cuvette, which shows that this P450 displays significant catalase activity; this enzyme also appears to be very stable to peroxide treatment. Treatment of OxyA_com_ with peroxide shows a similar trend of increased sensitivity to oxidative heme bleaching compared to OxyC_com_, although it is significantly more stable than OxyA_kis_. Neither of the complestatin P450s showed increase in the absorbance between 600 and 700 nm, which showed that the damage occurring in the OxyA_kis_ enzyme was unusual and, thus, worthy of further investigation, given the possible parallels to heme oxygenase chemistry in this case.

### EPR Measurements of OxyA_kis_ and OxyC_kis_ During Peroxide Treatment.

**FIGURE 9:**
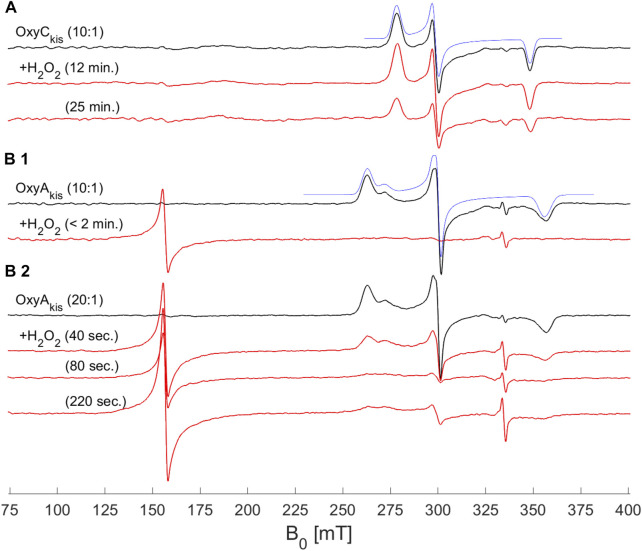
EPR spectra of OxyA_kis_ and OxyC_kis_ after addition of hydrogen peroxide. (X-band (9.383 GHz) CW EPR spectra measured at 7.5 K on OxyA_kis_ and OxyC_kis_ (concentration 200 μM, black lines) before and after addition of a 0.3% hydrogen peroxide solution for the indicated reaction times (red lines). **(A)** OxyC_kis_ after addition of H_2_O_2_ at a 10:1 volume ratio (protein:H_2_O_2_). **(B1)** OxyA_kis_ after addition of H_2_O_2_ at a 10:1 volume ratio. **(B2)** OxyA_kis_ after addition of H_2_O_2_ at a 20:1 volume ratio (protein:H_2_O_2_). The low-spin OxyC_kis_ spectrum is simulated with a single paramagnetic component (blue line) with g-values of *g* = (2.410, 2.245, and 1.924]) and the low-spin OxyA_kis_ spectrum with a two-component model, *g*_A_ = (2.551, 2.232, and 1.879) (77%) and *g*_B_ = (2.469, 2.249, and 1.893) (23%) (blue line). Several EPR components are often observed in the resting state of P450s, and this is attributed to different conformations of coordinated water/residue side chains in the active site. The large signal at ∼156 mT in the OxyA_kis_ data is assigned to non-heme iron. The small sharp signal at 335 mT is from an unassigned organic radical.

To shed further light on the mechanism of heme damage occurring in the treatment of OxyA_kis_ and OxyC_kis_ with hydrogen peroxide, we performed continuous wave (CW) electron paramagnetic resonance (EPR) spectroscopic experiments on these enzymes both before and after treatment with varying amounts of peroxide (Figure 9). Before treatment, both enzymes exhibit a low-spin (LS, *S* = ½) EPR signal characteristic of the Fe^3+^ ion of heme (Harbort et al., 2017). Upon addition of 20 μL of 0.3% H_2_O_2_ to 200 μL of 200 μM protein, OxyA_kis_ completely loses its LS heme signal in less than 2 min, and a large signal appears at 156 mT (*g* = 4.27), indicating that the iron has been removed from the heme and is now in a high-spin state (*S* = 5/2). By comparison—and even after 10 min of H_2_O_2_ exposure—OxyC_kis_ retains the same EPR signal as prior to peroxide addition, together with the same intensity (concentration of LS centers) within experimental error (∼10%). Furthermore, after 20 min, this LS EPR signal from the Fe^3+^ heme is reduced by only ∼50% (data not shown), showing yet again the increased stability of OxyC_kis_ to hydrogen peroxide when compared to the behavior of OxyA_kis_.

While these experiments did not provide supporting evidence for a distinct oxidized heme intermediate, they did indicate that the loss of heme from these proteins was *via* oxidative damage. Given that the intermediates involved in this oxidative process were clearly unstable, we next turned to high-resolution X-ray crystallography to assess the OxyA_kis_ active site and determine whether features in the heme environment could be implicated in the relative instability of this P450.

### Structural Characterization of OxyA_kis_

**FIGURE 10:**
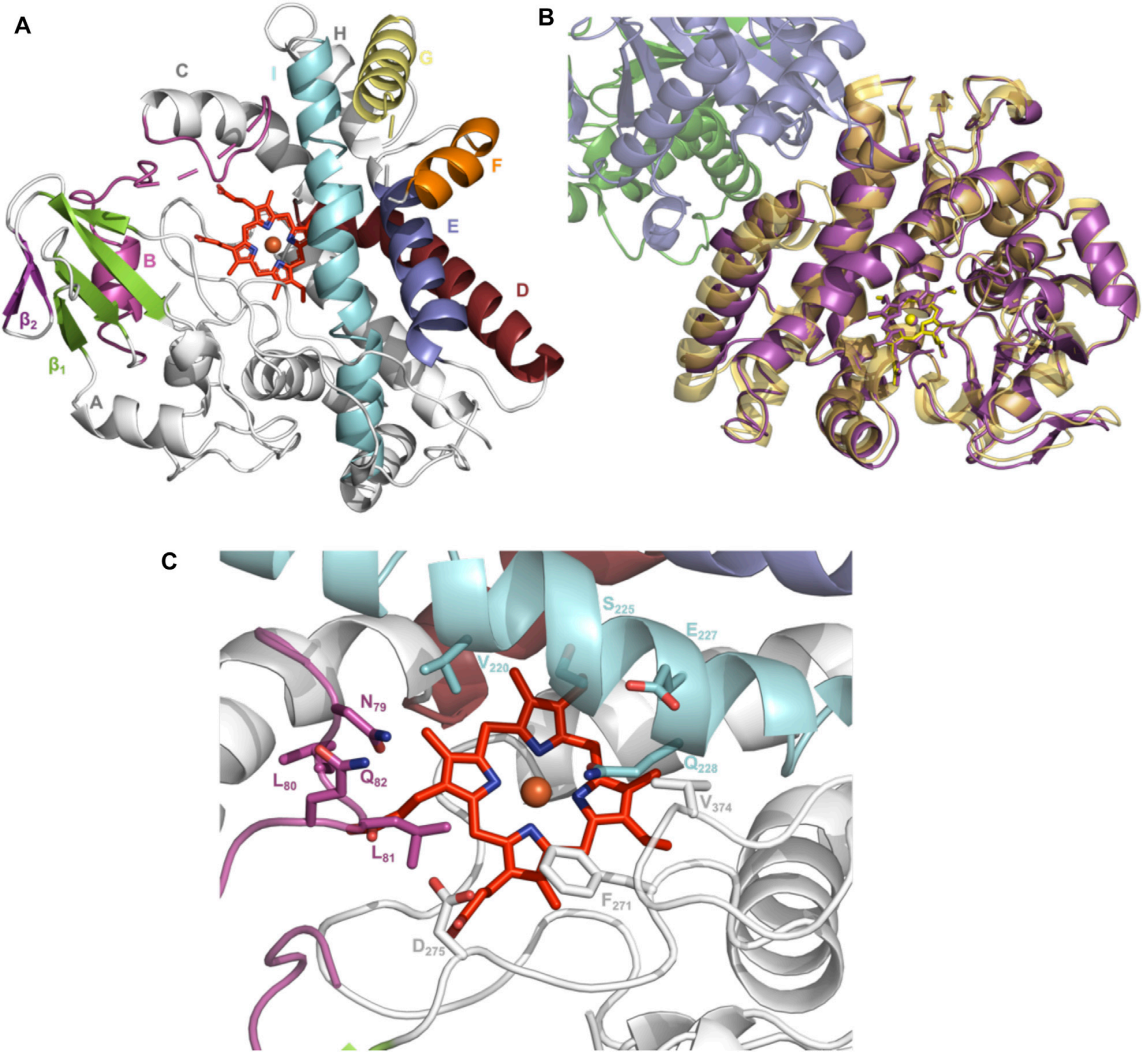
Structure of the P450 enzyme OxyA_kis_. **(A)** Overview of OxyA_kis_ structure. B-Helix and BC-loop shown in magenta, D-helix shown in firebrick red, E-helix shown in blue, F-helix shown in orange, G-helix shown in yellow, I-helix shown in cyan, β_1_ region shown in green, β_2_ region shown in purple, and typical heme orientation shown in red sticks. **(B)** Aligned structure of OxyA_kis_ overlaid with the previously solved OxyA_kis_/X-domain complex (PDB code: 6M7L). Yellow–OxyA_kis_; purple–previously solved OxyA_kis_ in complex with X-domain; green–X-domain N-terminal subdomain; blue–X-domain C-terminal subdomain. **(C)** OxyA_kis_ active site (side chains shown as sticks), coloring as in **(A)**.

To elucidate the structural implications of P450 inactivation *via* oxidative damage, we turned to X-ray crystallography. Prior to this study, we solved the structure of OxyA_kis_ in the complex with the NRPS recruitment X-domain from the last module of kistamicin NRPS to a resolution of 2.6 Å (Greule et al., 2019). This complex showed that the overall structure of OxyA_kis_ corresponded well to that reported for other Oxy enzymes and that the interface between the Oxy enzymes and X-domain was conserved to that reported in related systems. However, the resolution of this complex was insufficient to obtain a detailed understanding of the heme environment of this P450. Thus, we sought to crystallize OxyA_kis_ alone, and we were able to identify conditions in which the P450 would crystallize and diffract to high resolution (1.6 Å, Figure 10A, SI Supplementary Table S1).

With OxyA_kis_ no longer in complex with the X-domain, we observed some changes to the flexible regions of the P450, noting that the loop connecting the F and G helices could no longer be resolved due to lack of clear density and suggesting that this loop is stabilized in the complex. Additional gaps in the structure of mobile sites surrounding the heme and active site were present, suggesting that these regions are disordered without the bound substrate, which is in keeping with results from many structurally characterized P450s. Despite these minor differences, the overall fold of OxyA_kis_ remained largely identical to the structure previously solved in complex with the X-domain (RMSD = 0.47 Å, Figure 10B) (Greule et al., 2019).

**FIGURE 11:**
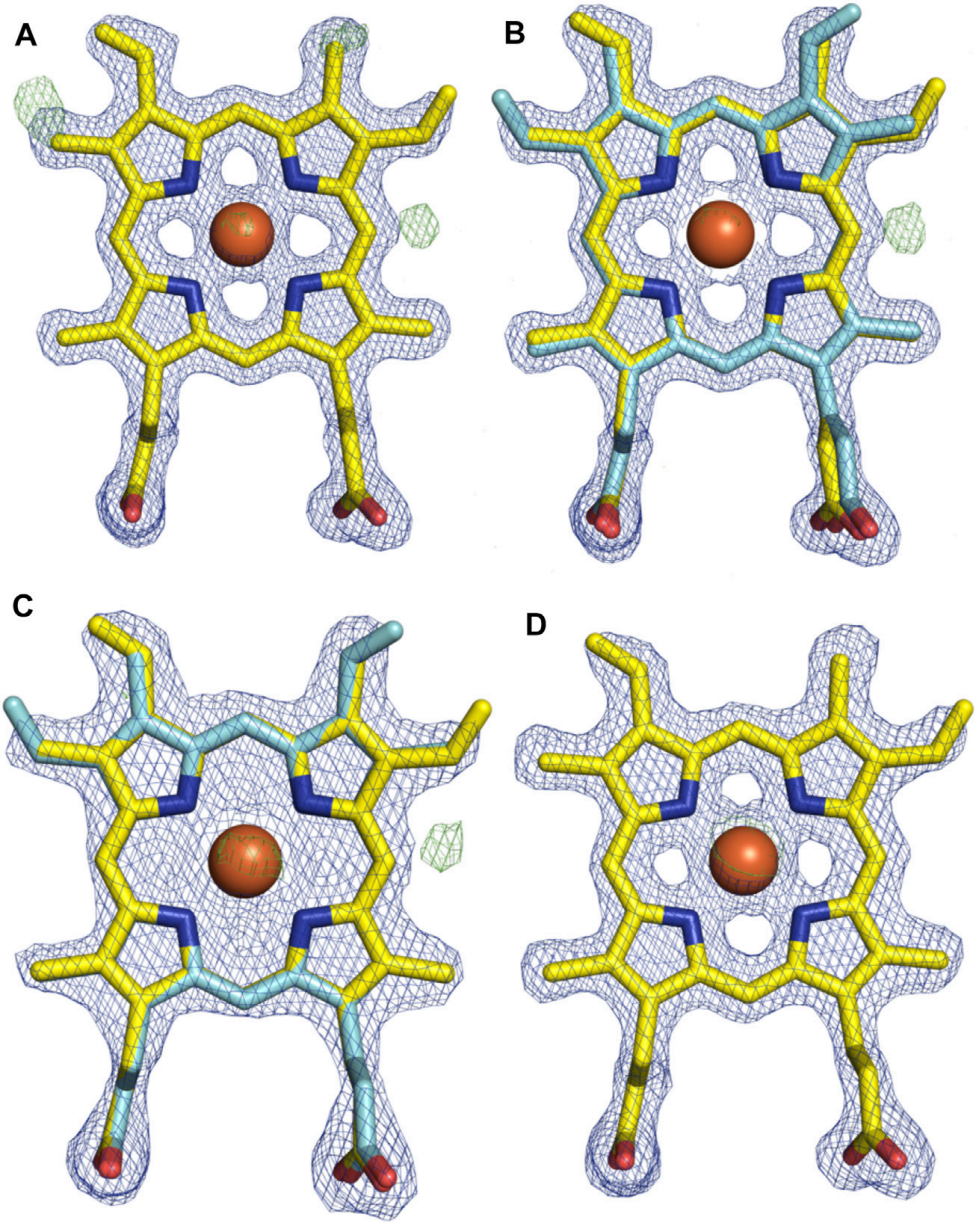
Comparison of the electron density around the heme moiety in OxyA_kis_ structure refinement using differing ratios of typical and “flipped” heme orientations. Electron density map (blue mesh; +1.5σ 2mFo-DFc) and difference map (green mesh; +3σ mFo-DFc) after refinement of fitted heme ligands. **(A)** Initial data set modeled with 100% typical heme orientation (yellow sticks). **(B)** Initial data set modeled with typical and flipped heme ligand fitted at a 2:1 ratio (0.67 and 0.33 occupancy). **(C)** Low-dose X-ray data collection modeled with typical and flipped orientation at the same ratio as above. **(D)** Subsequent protein batch with typical heme orientation fitted. Yellow–carbon atom, typical heme orientation; cyan–carbon atom, flipped conformation; orange–carbon atom, flipped conformation and modified heme; red–oxygen atom; blue–nitrogen atom; orange sphere–iron atom.

With the general fold essentially unchanged, we performed a detailed analysis of the active site of this P450, which revealed a mixed population of heme orientations as seen in the Fo–Fc difference map electron density at the substituent vinyl and methyl groups (Figures 11A,B). With few reported exceptions, P450s incorporate the heme moiety, such that the β-position of the heme is placed under the central I-helix, which means that the vinyl and methyl groups at positions 4/5 of the heme also reside under the I-helix (Rudolf et al., 2017). Much less common are examples of P450s in which the heme is inserted in a “flipped” orientation, meaning that the δ-position of the heme (and neighboring 1-methyl and 2-vinyl groups) is placed under the I-helix: examples of P450s displaying this heme orientation include CYP154A1 (PDB ID:1ODO) (Podust et al., 2004), SgvP (4MM0) (Li et al., 2017), CYP105P2 (5IT1) (Lee et al., 2016), StaF (PDB ID: 5EX8) (Ulrich et al., 2016), and OxyA_tei_ (PDB ID: 5HH3) (Haslinger and Cryle, 2016). In this context, CYP121A1 (PDB ID: 1N40) is the only example of a P450 showing a 50:50 mixture of the heme orientations within the active site (Leys et al., 2003). The reasons and mechanism responsible for this distinct preference of one heme orientation within P450s are not yet understood, and it is unclear whether the alternate heme orientation influences the catalytic activity of these enzymes. Two of the P450s with variant heme orientations are OxyA homologs from glycopeptide antibiotic (GPA) biosynthesis that catalyze oxidative phenolic coupling (OxyA_tei_ from teicoplanin biosynthesis and StaF from A47934 biosynthesis) (Haslinger and Cryle, 2016; Ulrich et al., 2016), while CYP121A1 catalyzes the related process of aryl crosslinking in mycocyclosin. Given that P450s from GPA biosynthesis are overrepresented in the structurally characterized P450s bearing the alternate heme conformation, we explored various ratios of the heme orientations that could model occupancies (2:1 ratio) of a flipped heme ligand in the structure of OxyA_kis_.

However, even once the density of the methyl/vinyl groups was well-explained by a mixture of heme orientations; there was still evidence for additional density at the side of the heme immediately below the I-helix (β-position, Figure 11C). Given that heme oxygenase chemistry exploits the hydroxylation of heme during its degradation, it is tempting to speculate that this density may represent hydroxylation of the heme (albeit, here the hydroxyl group would be located at a different position of the heme (β versus α position in the heme oxygenase reaction)). To explore the possibility of radiation damage to the heme due to long data collection causing this modification, we collected several datasets using an attenuated beam. Even with a beam attenuation of 95% and collecting data in small wedges to reduce possible damage, this dataset still showed density in the difference map for modification at the β-position of the heme (SI Supplementary Table S1).

With a possible correlation of the orientation of the heme moiety within the OxyA_kis_ active site with this unusual density, we next sought to determine if this was dependent on the orientation of the inserted heme. To carry out this, we crystallized OxyA_kis_ from additional expression batches and observed that the orientations of the heme ligand occupied were batch-specific. Furthermore, we identified a batch in which all proteins contained entirely the typical P450 heme conformation, from which we then solved a structure to a resolution of 1.8 Å (SI Supplementary Table S1). This structure was essentially identical to that of the isolated OxyA_kis_ structure we had solved initially (RMSD = 0.68) with the heme present in the active site in the typical orientation seen with most P450s, revealing that the orientation of the heme does not otherwise impact the fold of the P450s. A closer investigation of the heme moiety in this structure revealed no additional density present at the β-position, supporting a correlation between the additional density on the heme moiety and orientation of the heme itself.

### Improved OxyA_kis_ Stability is Enabled by Tyr99 to Phe Mutation

**FIGURE 12:**
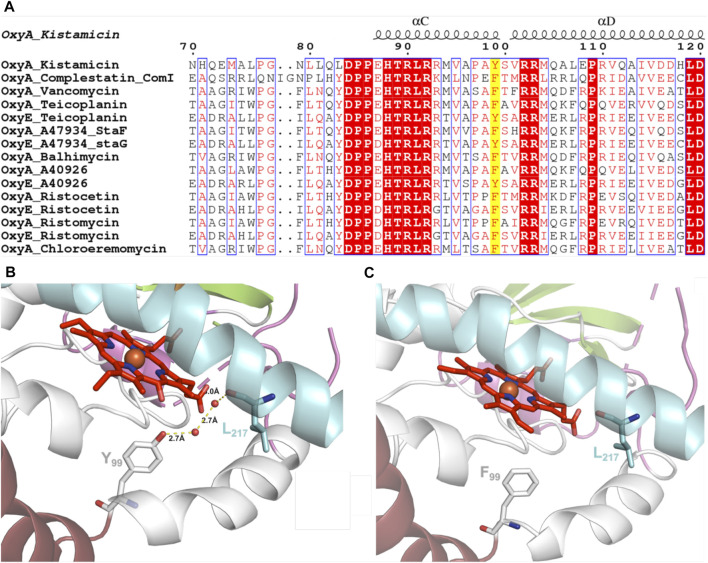
**(A)** Sequence alignment of OxyA and OxyE P450 enzymes from closely related GPAs (conserved residues highlighted in red, similar residues shown in red text, site of Tyr99Phe mutation highlighted in yellow; figure generated using ESPript (Robert and Gouet, 2014). **(B)** Wild-type OxyA_kis_ with water-mediated hydrogen bonding between Tyr99 and the I-helix residue Leu217 (water shown as red spheres and hydrogen bonding shown as yellow dashed lines with distances labeled). **(C)** OxyA_kis_ Tyr99Phe mutant with loss of coordinated water and hydrogen bonding; single I-helix conformation shown with flipped heme orientation omitted for clarity.

GPA crosslinking is implicated to occur *via* two 1-electron oxidation steps, which generate intermediate phenolic/indolic radical species (Ali et al., 2020). Given this, the potential for autooxidation of Tyr residues close to the P450 active site appears to be a particular challenge for this subclass of P450 enzymes to overcome. Having seen the extreme sensitivity of OxyA_kis_ for oxidative damage, we inspected the P450 active site for Tyr residues close to the heme to ascertain whether such residues could be playing a role in this sensitivity. The tyrosine residue at position 99 (position 119 in the His-tagged OxyA_kis_ protein) is oriented in such a way that the phenol moiety is very close to the site of additional density present in the OxyA_kis_ structure (distance from the phenol to the site of damage is 3.7 Å). This residue also appears to coordinate a water molecule [2.4 Å; 4 Å from the β-position; 2.7 Å to another molecule (water 202)]. Given this positioning, we performed the sequence alignment of multiple Oxy enzymes from different GPA pathways, which revealed that in most Oxy enzymes, the Tyr99 position is typically occupied by a phenylalanine residue instead (Figure 12A). Indeed, the presence of a tyrosine residue at this position is only observed in the OxyA_kis_ and in a group of closely related OxyE enzymes (OxyE_dbv_ from A40926 biosynthesis, OxyE_tei_ from teicoplanin biosynthesis, and OxyE_sta_ from A47934 biosynthesis), which are responsible for the installation of the additional F–*O*–G ring observed in teicoplanin-like GPAs (Cryle et al., 2011; Peschke et al., 2016c). However, in the structure of the only OxyE homolog to be crystallized, OxyE_tei_, different I-helix residues alter the environment of the equivalent Y99 residue, with Asn223 in OxyE_tei_ in hydrogen bonding distance to this phenol moiety (2.5 Å) compared to the equivalent residue in OxyA_kis_ (Val221, 4.1 Å from the phenol moiety) (Cryle et al., 2011).

**FIGURE 13:**
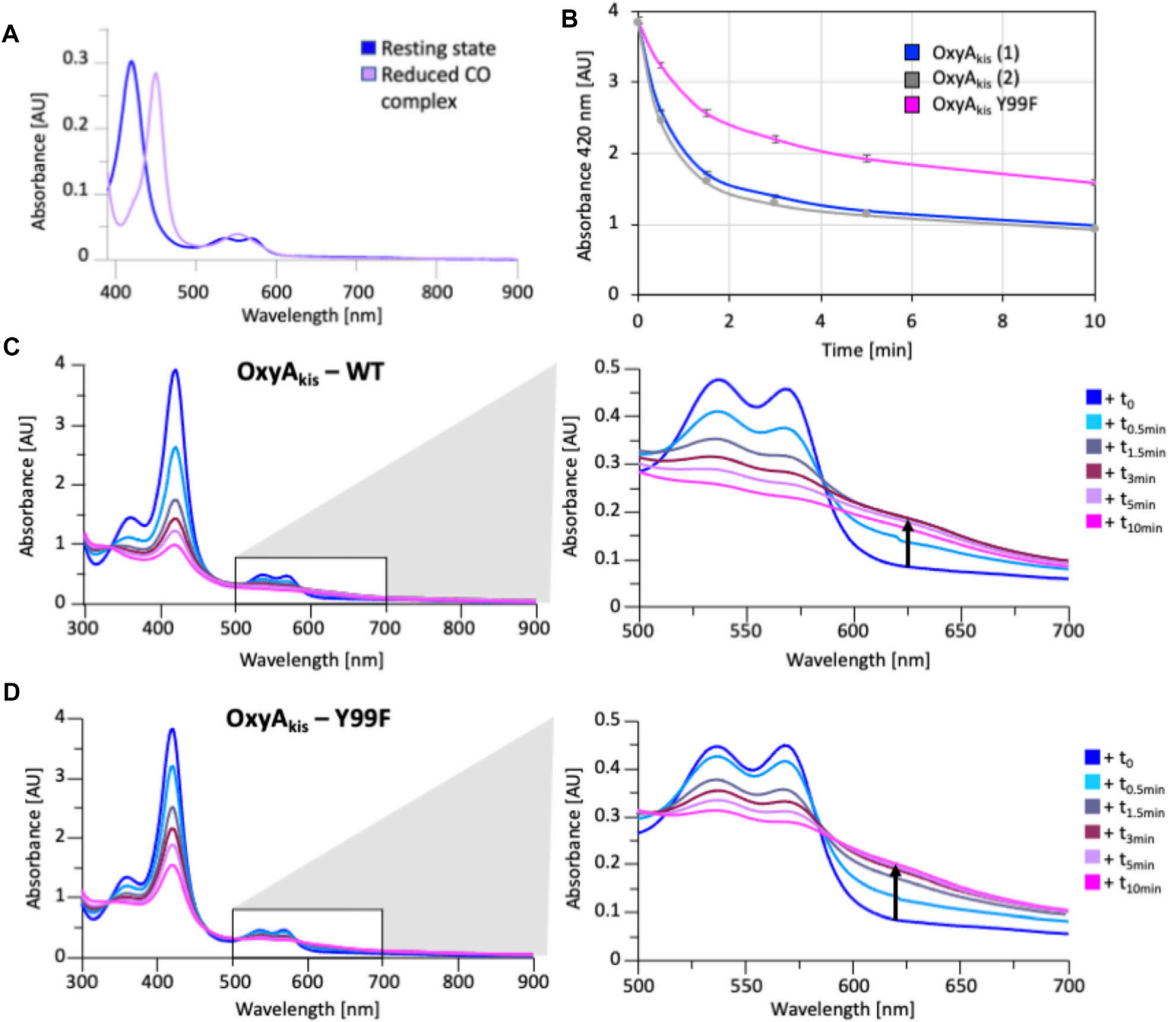
Characterization of OxyA_kis_ Tyr_99_Phe mutant. **(A)** UV/Vis spectra showing the conversion of the resting state spectra (blue) into the reduced CO difference spectra (mauve); the shift to 450 nm indicates the catalytic competence of the P450. **(B)** Rate of bleaching of the heme Soret peak (420 nm) of OxyA_kis_ upon peroxide addition of expression batches 1 (blue) and 2 (gray) of the WT proteins plus the Y_99_F mutant (pink). **(C)** UV/Vis spectra of OxyA_kis_ after addition of 0.4 mM hydrogen peroxide at different time points. UV/visible spectra shown from 390 to 900 nm (left panel) and a zoomed view of 500–800 nm (center panel). The shoulder in absorbance between 600 and 700 nm is indicated by an arrow. **(D)** UV/Vis spectra of the OxyA_kis_ Y_99_F mutant after addition of 0.4 mM hydrogen peroxide at different time points. UV/visible spectra shown from 390 to 900 nm (left panel) and a zoomed view of 500–800 nm (center panel). The shoulder in absorbance between 600 and 700 nm is indicated by an arrow.

As the phenol group present in Tyr99 appeared to be appropriately positioned to play a role in potential oxidative heme damage in the catalytic site of OxyA_kis_, we next cloned a mutant in which Tyr_99_ was mutated into Phe by site-directed mutagenesis. Verification of the mutation by DNA sequencing and protein mass spectrometry following the expression and purification of the mutant protein indicated that the mutation was present in this protein (SI Supplementary Figure S4). The reduced, CO-bound spectra of the OxyA_kis_-Y99F mutant enzyme showed that the heme environment of the mutant was preserved and that the enzyme was catalytically competent, with a high proportion of the 450 versus 420 nm species present (Figure 13). The addition of H_2_O_2_ revealed that the mutant enzyme was slightly more stable to oxidative damage than the wild-type enzyme, although the mutant still exhibits the shoulder between 600 and 700 nm and is more sensitive to oxidizing agents than OxyC_kis_ (Figure 7).

To examine this mutant further, OxyA_kis_-Y_99_F was crystallized under the same conditions used for the native OxyA_kis_. These crystals, which are diffracted to a resolution of 1.8 Å (Figure 12B, SI Supplementary Table S1), showed a mixed population of heme orientations in the enzyme active site (6:1 normal orientation/flipped orientation). Interestingly, no additional density has been observed at the β-position of the heme moiety as had been previously observed with the WT crystals. We observed weak and somewhat distorted electron density for the mutated phenylalanine residue itself, suggesting that the side chain exists in multiple conformations in the structure (SI Supplementary Figure S5). Furthermore, it appears that residues 223–228 of the I-helix now adopt two conformations above the heme moiety. In the wild-type structure, a network of hydrogen bonding is observed between the Tyr99 phenol moiety and amide backbone of the I-helix residue Leu217, mediated *via* two water molecules (Figure 12C). The Tyr99Phe mutation results in the loss of this H-bond network (Figure 12D), which we then attribute to the differences seen in the structure by permitting both the increase in flexibility of the Phe99 side chain and further the second conformation adopted by the I-helix. While unusual, the CO spectra of the mutant shows that it retains the heme thiolate ligand, suggesting that this alternate conformation of the I-helix does not appear to dramatically influence the incorporation of the heme moiety.

### Tripeptide Cyclization Using OxyA_kis_

Having seen that OxyA_kis_ is, indeed, highly susceptible to oxidative damage, we finally sought to reconstitute activity from this enzyme in a manner that would provide greater probability for success. The natural peptide substrate of OxyA_kis_ is both hydrophobic and requires prior OxyC_kis_ activity to insert the C–*O*–D ring, so we turned to the approach first pioneered by Robinson et al., in their studies with OxyB_van_ and sought to directly cyclize minimal PCP-X–bound tripeptides (Woithe et al., 2007; Woithe et al., 2008), given that our experiments with multienzyme cascades and complex peptide substrates had previously failed. To this end, we synthesized a panel of seven tripeptide CoAs based on common residues found in Oxy-mediated crosslinks (Hpg, Tyr, and Trp) in positions 1 and 3 of the peptide together with variable residues in position 2 (Phe, Phg, and Hpg) of these peptides (**1–7**); the effect of altering the stereochemistry of positions 2 and 3 in the peptide was also explored. Following their synthesis and enzymatic loading onto a PCP-X_kis_ construct using the promiscuous transferase Sfp, we next subjected these seven peptides to enzymatic turnover by both OxyC_kis_ and OxyA_kis_ (Table 1). As had been anticipated based on previous results, OxyC_kis_ displayed high (>70%, **5**, **6**) and moderate turnover (>25%, **1**, **7**) of four peptides, with only trace modification of the remaining peptides (**2**, **3**, **4**). In comparison, OxyA_kis_ (new batch, single anticipated heme geometry present) displayed no activity toward four of the peptides, including the Trp-containing peptide. Gratifyingly, however, cyclization activity was observed to low levels with **1** and moderate levels with peptides **5** and **6**. These peptides mimic the stereochemistry of the residues involved in the natural ring formed by OxyA_kis_ and further show that while the natural Trp residue is not accepted (possibly due to lack of steric restraint in these tripeptides as would be seen in the biosynthetic monocyclic heptapeptide), OxyA_kis_ can instead install aromatic crosslinks between phenol-containing aromatic side chains. This supports the versatility of such P450 enzymes for aromatic crosslinking between a range of possible side chains (demonstrated in the OxyC_corb_ cyclization of Tyr–Hpg instead of Hpg–Hpg in the final OxyC-mediated crosslink) (Culp et al., 2020), which can also lead to different crosslinked species in these peptides (as seen with OxyC_kis_-mediated insertion of both phenolic and aryl crosslinks) (Greule et al., 2019). Such versatility helps explain why the OxyA and OxyE enzymes found in the corbomycin gene cluster can install different crosslinks (an aryl Trp–Hpg vs. phenolic Dpg–Hpg crosslink, respectively) despite the sequence similarity they display. These results demonstrate that the reconstitution of type V GPA OxyA enzymes *in vitro* is feasible—albeit challenging—and that this could be investigated for chemically synthesized monocyclic peptide substrates in future experiments. Furthermore, they indicate that the roles for such P450s in engineered complex peptide crosslinking pathways could well be more flexible than has perhaps seemed likely to date, opening further opportunities to exploit such enzymes as diverse biocatalysts.

**TABLE 1:** Structure of tripeptide substrates **1–7** and the results of cyclization assays performed using the kistamicin, OxyC, and OxyA enzymes.

Peptide	AA_1_ residue (N) identity	AA_2_ residue identity	AA_3_ residue (C) identity	OxyC_kis_ cyclization^b^	OxyA_kis_ cyclization^b^
**1**	(D)-Hpg	(D)-Phe	(D)-Hpg	31%	8%
**2**	(D)-Hpg	(D)-Phe	(L)-Hpg	Trace	—
**3**	(D)-Hpg	(L)-Phe	(L)-Hpg	Trace	—
**4**	(D)-Hpg	(D)-Phg	(D)-Hpg	Trace	—
**5**	(D)-Tyr	(D)-Hpg	(D)-Hpg	85%	46%
**6**	(D)-Tyr	(L)-Hpg	(D)-Hpg	71%	35%
**7** ^a^	(D)-Trp	(D)-Hpg)-Hpg	30%	—	—

a Natural kistamicin mimic [residue 2 altered from (D)-Dpg to (D)-Hpg due to lack of commercial availability].

b Proportion of the cyclized peptide based on the HRMS analyses; percentage calculated by dividing the peak area of the cyclic peptide product by the total peak area of (starting material plus product). Hydrolysis of the peptide product was low (<5% in all cases); hence, only the methylamide products generated through methylamine workup of the turnover reactions are included here.

## Conclusion

Structural and biochemical analysis of OxyA_kis_ has shown that this P450 enzyme is highly prone to oxidation and can display an atypical heme orientation in different protein batches. Crystallographic experiments revealed additional unexplained density adjacent to the heme moiety in structures where this alternate heme conformation was present, suggesting that P450s may prefer their typical heme orientation to avoid potential oxidative damage. Turnover experiments with OxyA_kis_ have shown that it is possible to reconstitute peptide crosslinking activity from this enzyme, although it remains highly sensitive to oxidation and unsuitable for inclusion in multienzyme cascades. This is a challenge that needs to be overcome to explore both the selectivity of this central Type V GPA crosslinking enzyme and exploit this crosslinking for the generation of diverse crosslinked GPA peptides *in vitro*. Given that GPA homologs are known to exhibit significantly different activity on reconstitution, this suggests that further examples of OxyA enzymes from Type V systems should be sought and analyzed to identify candidates for future research concerning the GPA cyclization cascade.

## Data Availability

The datasets presented in this study can be found in online repositories. The names of the repository/repositories and accession number(s) can be found at http://www.wwpdb.org/, 7TTA, 7TTB, 7TTO, 7TTP, 7TTQ; http://www.proteomexchange.org/, PXD030867.
