# Supplementary material for: The Cytochrome P450 OxyA from the Kistamicin Biosynthesis Cyclization Cascade is Highly Sensitive to Oxidative Damage
Source: Front Chem. 2022 Apr 8;10:868240. doi: 10.3389/fchem.2022.868240 (PMC9023744; doi:10.3389/fchem.2022.868240)
Supplement: Supplementary file 1 [file DataSheet1.pdf]

The Cytochrome P450 OxyA from the kistamicin biosynthesis cyclization cascade is highly sensitive to oxidative damage

Anja Greule,[1,2,†] Thierry Izoré,[1,2,†] Daniel Machell,[1,2,3] Mathias H. Hansen,[1,2,3] Melanie Schoppet,[1,2] James J. De Voss,[4] Louise K. Charkoudian,[5] Ralf B. Schittenhelm,[1,6] Jeffrey R. Harmer,[7] and Max J. Cryle [1,2,3]*

1. The Monash Biomedicine Discovery Institute, Department of Biochemistry and Molecular Biology, Monash University, Clayton, Victoria 3800, Australia.
2. EMBL Australia, Monash University, Clayton, Victoria 3800, Australia.
3. ARC Centre of Excellence for Innovations in Peptide and Protein Science, Clayton, Victoria 3800, Australia.
4. Centre for Advanced Imaging, The University of Queensland, St Lucia, Queensland 4072, Australia.
5. Department of Chemistry, The University of Queensland, St Lucia, Queensland 4072, Australia.
6. Department of Chemistry, Haverford College, Haverford, PA 19041, USA.
7. Monash Proteomics and Metabolomics Facility, Monash University, Clayton, VIC, Australia.

† Authors contributed equally.

Supporting Information

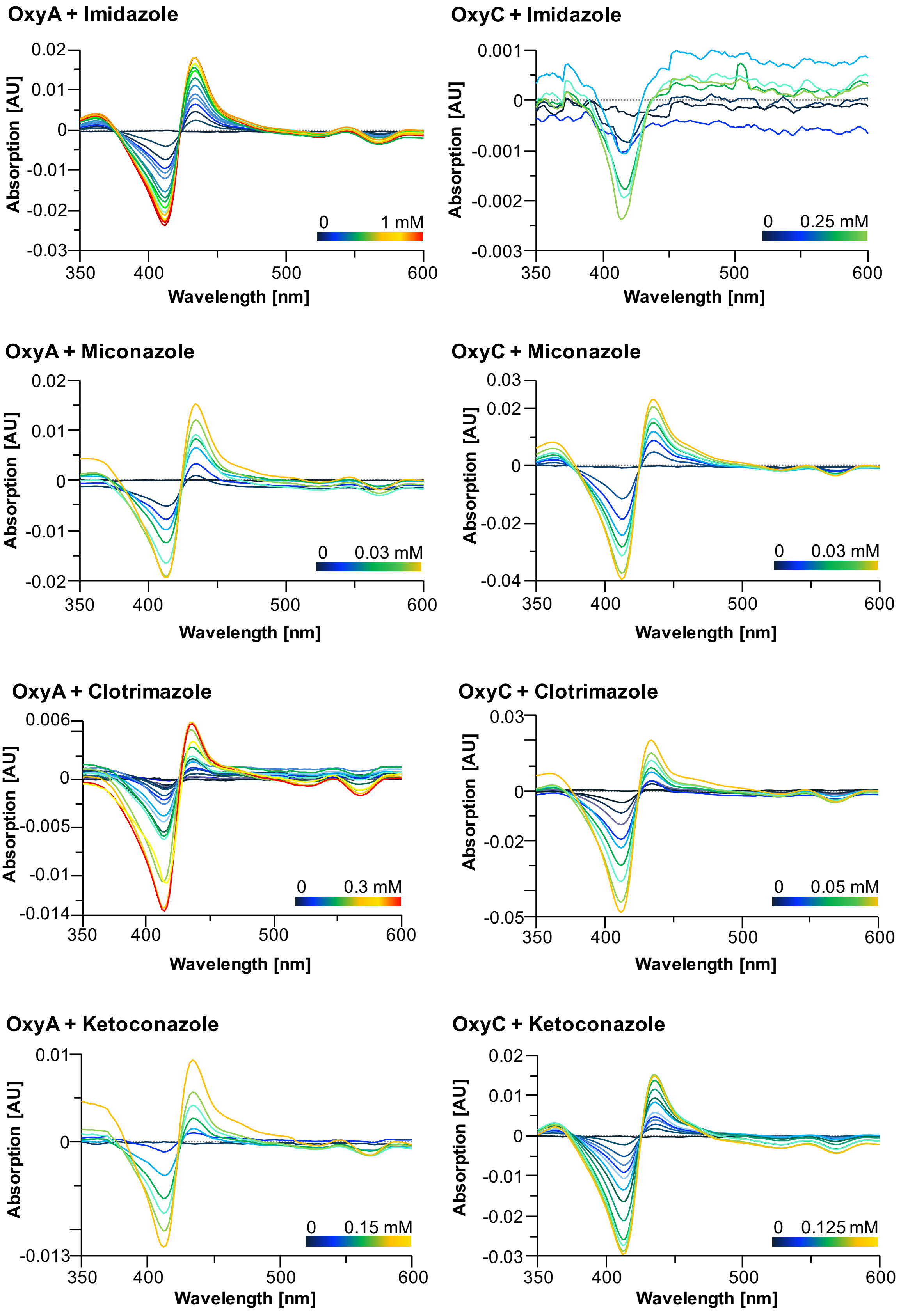
OxyA + Imidazole
Absorption [AU]
Wavelength [nm]
0 1 mM
OxyC + Imidazole
0 0.25 mM
OxyA + Miconazole
0 0.03 mM
OxyC + Miconazole
0 0.03 mM
OxyA + Clotrimazole
0 0.3 mM
OxyC + Clotrimazole
0 0.05 mM
OxyA + Ketoconazole
0 0.15 mM
OxyC + Ketoconazole
0 0.125 mM

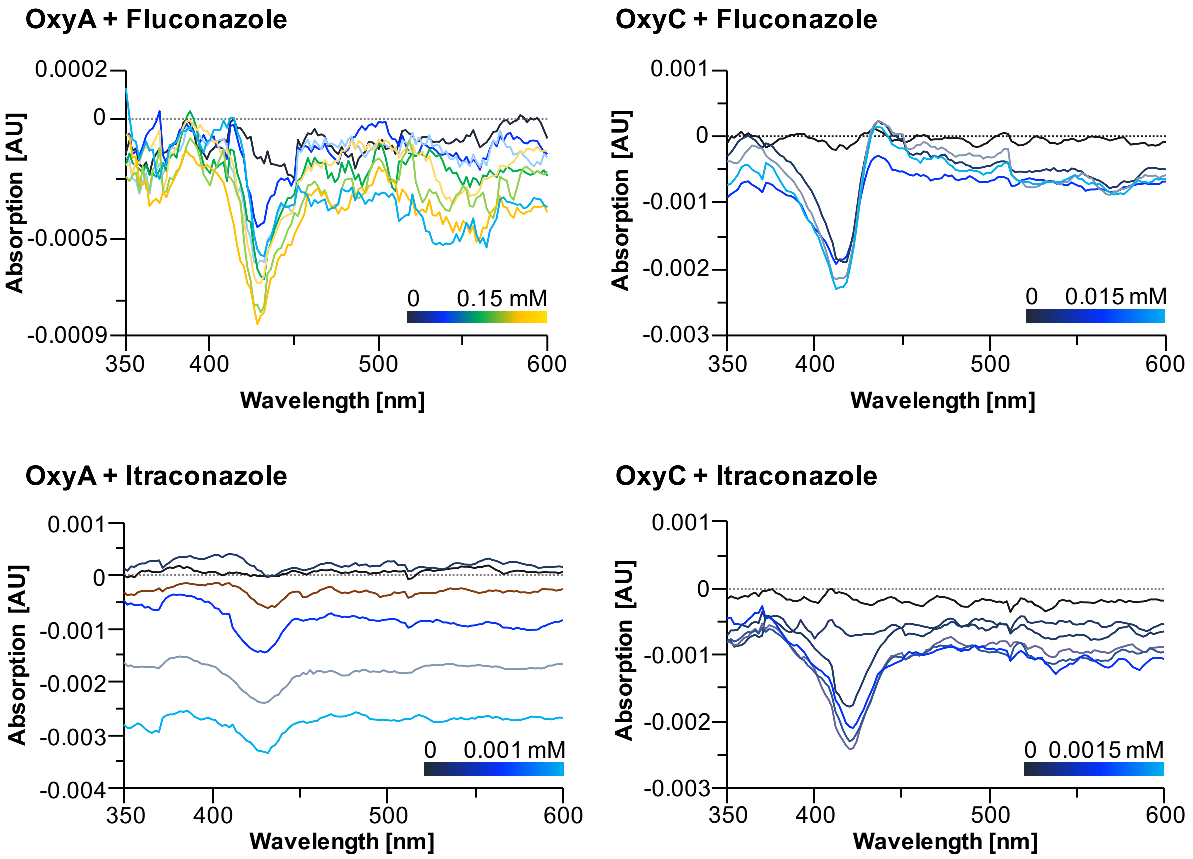

Figure S1. UV-visible spectroscopic binding studies of $OxyA_{kis}$ and $OxyC_{kis}$ binding to different azole inhibitors. $OxyA_{kis}$ (left) and $OxyC_{kis}$ (right) were incubated with different amounts of azole inhibitor and the UV-vis spectra was measured in comparison to the control + DMSO between 350 – 600 nm. Inhibitors tested were imidazole, miconazole, clotrimazole, ketoconazole, fluconazole and itraconazole.

Table S1. Data collection and refinement statistics (molecular replacement)

	**$OxyA_{kis}$ Mixed heme population**	**$OxyA_{kis}$ Mixed heme pop., attenuated beam**	**$OxyA_{kis}$ Normal heme**	**$OxyA_{kis}$ Normal heme, imidazole complex**	**$OxyA_{kis}$ Y99F mutant**
**PDB Code**	7TTO	7TTA	7TTP	7TTQ	7TTB
**Data collection**					
Space group	$P3_221$	$P3_221$	$P3_221$	$P3_221$	$P3_221$
Cell dimensions					
a, *b*, *c* (Å)	70.0, 70.0, 132.4	70.1, 70.1, 132.7	69.8, 69.8, 132.5	69.8, 69.8, 132.4	69.7, 69.7, 132.4
α, β, γ (°)	90, 90, 120	90, 90, 120	90, 90, 120	90, 90, 120	90, 90, 120
Resolution (Å)	44.13 - 1.60	44.77 - 2.00	35.66 - 1.80	35.64 - 1.80	35.61 - 1.62
R_{merge}	0.014 (0.134)	0.053 (0.448)	0.021 (0.259)	0.025 (0.397)	0.020 (0.231)
$I / \sigma I$	25.18 (4.53)	8.10 (1.56)	23.42 (2.99)	16.06 (1.76)	18.29 (2.64)
$CC_{1/2}$	1 (0.96)	1 (0.78)	1 (0.85)	1 (0.72)	1 (0.85)
Completeness (%)	99.5 (98.7)	98.5 (95.3)	94.2 (96.7)	99.9 (100.00)	95.8 (57.4)
Redundancy	2.0 (2.0)	1.9 (1.8)	2.0 (2.0)	2.0 (2.0)	2.0 (2.0)
**Refinement**					
Resolution (Å)	44.13 - 1.60	44.77 - 2.00	35.66 - 1.80	35.64 - 1.80	35.61 - 1.62
No. reflections	100474 (9792)	48925 (4450)	65368 (6630)	70380 (6931)	91786 (5325)
R_{work} / R_{free}	0.20 / 0.23	0.18 / 0.23	0.19 / 0.22	0.19 / 0.22	0.18 / 0.20

No. atoms					
Protein	2787	2761	2674	2840	2784
Ligands	146	160	87	160	97
Water	229	229	218	168	222
B-factors					
Protein	28.21	37.07	27.72	30.31	25.38
Ligands	15.48	26.99	18.79	20.82	17.94
Water	31.47	39.23	32.07	31.92	31.71
R.m.s. deviations					
Bond lengths (Å)	0.002	0.012	0.009	0.005	0.010
Bond angles (°)	0.50	1.29	0.89	0.79	1.05

```
OxyAkis      MVAPEHRVLHLRDRLDLAAELKLLCERGPLVRIPLEDGS---AVHWFALGYDVVREVLGS   57
ComI         MASRDVPVYNRRDRLDPVPELVELRNRCPVLRTELHGGPSSQVVGWLVTGIDESREVLSD   60
             *.: :  * : ***** . **  * :* *::*  *..*    .* *:. * *  ****..

OxyAkis      EKFDKRVI--GTHFNHQEMALPGNLLQLDPPEHTRLRRMVAPAYSVRRMQALEPRVQAIV   115
ComI         QHRFTMLPPADTEAQSRRLQNIGNPLHYDPPEHTRLRKMLNPEFTMRRLRRLQPRIDAVV   120
             ::  . :   .*. : :.:   ** *: *********:*: * :::**:: *:**::*:*

OxyAkis      DDHLDTMASTGPPVEFLREVAGPMAARVACEFLGIPLDDRGELIRLTA----HRGGKRRR   171
ComI         EECLDAMEQAGAPADLMQHFAWQIPGHTACELLGVPRDDRAELSRHLDITRDDGRGRARQ   180
             :: **:* .:* *.::::..*  : .:.***:**:* ***.** *       .  *: *:

OxyAkis      VLNGHAYLAYMRELAARLRRDPGDGMLGMVARDHGADISDEELAGLCAVVMNSSVEQTES   231
ComI         MAAGRAYRAYFHQLTARQRRDPGDDLLGMLVREYGDEITDEELEGLAASLTSAGIENVAS   240
             :  *:** **:::*:** ******.:***:.*::* :*:**** **.* : .:.:*:. *

OxyAkis      CLAAGTLLLLEHPEQFALLRERPELGEQAVEEIVRYLSVFEGLDPRTATEDVEIGGQVIK   291
ComI         MLGLGTLVLLEHPDQLAELREKPELIDRAVEELLRHVSVIPTLSPRTALEDVPLGGHVVP   300
              *. ***:*****:*:* ***:*** ::****::*::**:  *.**** *** :**:*:

OxyAkis      KGEAVFCSLLAANRA----DPALDGFDITRKESRHVAFGHGIHHCLGAPLARMELRIAFT   347
ComI         KGERVICSAFAANRIATPGDDLEDGFDITREPAPHMAFGHGVHHCLGAPLARMQLRTAYQ   360
             *** *:** :****     *   *******: : *:*****:***********:** *:

OxyAkis      TLVSRFPSLRTAVPAEEIRFRPPSSNVFTLLELPLTW 384
ComI         ALWRRFPELRLAVPHEEIRFRMPSSRVYSVDALPVAW 397
             :*  ***.** *** ****** ***.*:::  **::*
```

Figure S2. OxyA$_{kis}$ / ComI alignment. [a,b]

[a] Generated with Clustal Omega[1]

[b] 49.2% Identity, Y99 position in OxyA$_{kis}$ / equivalent position highlighted in yellow

```
OxyCkis      MTDVTPGFVVDEATQVVTPEPDFII-TRKHLEPTDSLRALRKRGALLKINGHALGSLDVD   59
ComJ         MPQQAQRQAPQQQPRAQQAYPELLYTRRTRFDPADDLRAAPPLS------RYVIG--PNE   52
             * : :   . ::  :.    *:::   *.:::*:*.***    .       :.:*    :

OxyCkis      GTAYIWLATGYEVVRRILGDHENFSTRRRLTAGEPIDGTGVTPVKELVGHLMNLDPPEHT   119
ComJ         SDEWVWLATGYTEVRRILGDHTNFSTRRRWGAEGPNW-----RPPELVGHLMDYDPPEHT   107
             .  ::******  ******** *******  *  *          *******: ******

OxyCkis      RLRRMLTPEFTLRRIRRLEPVISEIIEDHLNLIEDAGPPADLQSMYAEPVGGATLCELIG   179
ComJ         RLRQMLTPEFTVRRLRRLEPDITAIIEEHLDTVEATGPGADLMPLFAQPVPGEVLCELIG   167
             ***:*******:**:***** *: ***:**: :* :** ***  ::*:** * .******

OxyCkis      VPRDDRAEFLRRCGLHLDMSRGSKRRAADSMAFNGYLDHLIGLQRKRPDDGFIGMLVQEH   239
ComJ         VPRDDRPEFLRHCHRHLDFSRSRKVRAADGAAFSRYLVSMVARQRKDPDDGFIGALVREH   227
             ****** ****:*  ***:**. * ****. **. **  ::. *** ******* **:**

OxyCkis      GDDVTDDELRGMITVVLLAGLDNISGMLGLGVLALLEHPEQIPVIFEERA----------   289
ComJ         GDDFTDEEMRGVCVLLILAGIDNIEGMIGLGVLAMLENPDQLPLLLGERDSTGGPGAGKG   287
             ***.**:*:**: .:::***:***.**:******:**:*:*:*::: **

OxyCkis      -----VTDRAVDELTRYLSITFQPTPRMALNDVVVDGQTIKAGEIVVCSLPMANRDEALT   344
ComJ         DGGRLASDRALDELIRYMSVANAPTPRTAVNDVRIGDQLIKAGETVICSLTMANRDPALT   347
                  .:***:*** **:*::  **** *:*** :..* ***** *:*** ***** ***

OxyCkis      PDPDVLDLRRQLAGHVGFGHGVHHCLGSSVSRVVLRLAYQALWRRFPDLRLAVPPEDIVF   404
ComJ         DGPDRLDLAREPVAHVAFGHGVHHCLGAALARTELRIAYKALWRRFPELRLAVPVEEVRF   407
              .** *** *: ..**.**********::::*. **:**:*******:****** *:: *

OxyCkis      -RKAITHGPKRLPVTWGPSAATR      426
ComJ         YNRALAHGVHRLPVAW-------      423
              .:*::** :****:*
```

Figure S3. $OxyC_{kis}$ / ComJ alignment. [a,b]

[a] Generated with Clustal Omega[1]

[b] 57.4% Identity, position equivalent to Y99 position in $OxyA_{kis}$ highlighted in yellow

**Score:**	4145
**Nominal mass (M_r):**	45178
**Calculated pI:**	6.26

Sequence similarity is available as an NCBI BLAST search of OxyA_Y119F against nr.

Search parameters

**MS data file:**	C:\MGFs\037\Tyr119Phe_1-A,6_01_16991.mgf
**Enzyme:**	semiTrypsin: cuts C-term side of KR unless next residue is P.<br>Cleavage is semi-specific. (Peptide can be non-specific at one terminus only.)
**Fixed modifications:**	Carbamidomethyl (C)

Protein sequence coverage: 86%

Matched peptides shown in ***bold red***.

```
  1 MGSSHHHHHH SSGLVPRGSH MVAPEHRVLH LRDRLDLAAE LKLLCERGPL
 51 VRIPLEDGSA VHWFALGYDV VREVLGSEKF DKRVIGTHFN HQEMALPGNL
101 LQLDPPEHTR LRRMVAPAFS VRRMQALEPR VQAIVDDHLD TMASTGPPVE
151 FLREVAGPMA ARVACEFLGI PLDDRGELIR LTAHRGGKRR RVLNGHAYLA
201 YMRELAARLR RDPGDGMLGM VARDHGADIS DEELAGLCAV VMNSSVEQTE
251 SCLAAGTLLL LEHPEQFALL RERPELGEQA VEEIVRYLSV FEGLDPRTAT
301 EDVEIGGQVI KKGEAVFCSL LAANRADPAL DGFDITRKES RHVAFGHGIH
351 HCLGAPLARM ELRIAFTTLV SRFPSLRTAV PAEEIRFRPP SSNVFTLLEL
401 PLTW
```

Unformatted sequence string: 404 residues (for pasting into other applications).

Sort peptides by ◉ Residue Number ○ Increasing Mass ○ Decreasing Mass

Query	Start – End	Observed	Mr(expt)	Mr(calc)	ppm	M	Score	Expect	Rank	U	Peptide
338	2 – 17	590.2931	1767.8574	1767.8414	9.01	0	48	0.021	1	U	M.GSSHHHHHHSSGLVPR.G
339	2 – 17	442.9716	1767.8574	1767.8414	9.03	0	37	0.26	1	U	M.GSSHHHHHHSSGLVPR.G
340	2 – 17	590.3054	1767.8945	1767.8414	30.0	0	9	1.7e+002	1	U	M.GSSHHHHHHSSGLVPR.G
341	2 – 17	442.9810	1767.8949	1767.8414	30.3	0	3	7.1e+002	3	U	M.GSSHHHHHHSSGLVPR.G
342	2 – 17	590.3077	1767.9013	1767.8414	33.9	0	9	1.9e+002	1	U	M.GSSHHHHHHSSGLVPR.G
343	2 – 17	442.9830	1767.9028	1767.8414	34.7	0	9	1.6e+002	1	U	M.GSSHHHHHHSSGLVPR.G
61	10 – 17	426.7366	851.4587	851.4613	-3.10	0	35	0.7	1		H.HSSGLVPR.G
160	18 – 27	560.7738	1119.5330	1119.5244	7.75	0	46	0.048	1	U	R.GSHMVAPEHR.V
161	18 – 27	374.1850	1119.5333	1119.5244	7.96	0	37	0.38	1	U	R.GSHMVAPEHR.V
162	18 – 27	560.7739	1119.5333	1119.5244	7.96	0	46	0.047	1	U	R.GSHMVAPEHR.V
163	18 – 27	374.1853	1119.5340	1119.5244	8.65	0	49	0.025	1	U	R.GSHMVAPEHR.V
178	33 – 42	572.3313	1142.6481	1142.6295	16.3	1	51	0.012	1	U	R.DRLDLAAELK.L
179	33 – 42	572.3347	1142.6548	1142.6295	22.2	1	51	0.012	1	U	R.DRLDLAAELK.L
180	33 – 42	572.3363	1142.6580	1142.6295	25.0	1	47	0.034	1	U	R.DRLDLAAELK.L
181	33 – 42	572.3364	1142.6582	1142.6295	25.1	1	41	0.12	1	U	R.DRLDLAAELK.L
182	33 – 42	572.3369	1142.6592	1142.6295	26.0	1	48	0.026	1	U	R.DRLDLAAELK.L
183	33 – 42	572.3374	1142.6603	1142.6295	26.9	1	38	0.23	1	U	R.DRLDLAAELK.L
184	33 – 42	572.3378	1142.6611	1142.6295	27.7	1	51	0.012	1	U	R.DRLDLAAELK.L
185	33 – 42	572.3380	1142.6615	1142.6295	28.0	1	31	1.4	1	U	R.DRLDLAAELK.L
186	33 – 42	572.3384	1142.6623	1142.6295	28.7	1	47	0.033	1	U	R.DRLDLAAELK.L
187	33 – 42	572.3387	1142.6629	1142.6295	29.2	1	44	0.068	1	U	R.DRLDLAAELK.L
188	33 – 42	572.3390	1142.6635	1142.6295	29.7	1	29	1.8	1	U	R.DRLDLAAELK.L
189	33 – 42	572.3393	1142.6641	1142.6295	30.3	1	32	0.98	1	U	R.DRLDLAAELK.L
190	33 – 42	572.3399	1142.6653	1142.6295	31.3	1	41	0.12	1	U	R.DRLDLAAELK.L
70	35 – 42	436.7635	871.5125	871.5014	12.7	0	41	0.16	1	U	R.LDLAAELK.L
136	53 – 62	519.2688	1036.5230	1036.5189	3.92	0	26	4.6	1	U	R.IPLEDGSAVH.W
263	53 – 64	685.8649	1369.7152	1369.6667	35.5	0	49	0.024	1	U	R.IPLEDGSAVHWF.A
297	53 – 66	777.9240	1553.8334	1553.7878	29.3	0	15	53	1	U	R.IPLEDGSAVHWFAL.G
415	53 – 72	748.7423	2243.2050	2243.1375	30.1	0	70	0.00011	1	U	R.IPLEDGSAVHWFALGYDVVR.E
416	53 – 72	748.7443	2243.2110	2243.1375	32.7	0	55	0.0033	1	U	R.IPLEDGSAVHWFALGYDVVR.E
417	53 – 72	748.7444	2243.2114	2243.1375	32.9	0	53	0.005	1	U	R.IPLEDGSAVHWFALGYDVVR.E
418	53 – 72	748.7452	2243.2137	2243.1375	33.9	0	38	0.17	1	U	R.IPLEDGSAVHWFALGYDVVR.E
419	53 – 72	748.7455	2243.2147	2243.1375	34.4	0	31	0.77	1	U	R.IPLEDGSAVHWFALGYDVVR.E
420	53 – 72	748.7455	2243.2148	2243.1375	34.5	0	57	0.0022	1	U	R.IPLEDGSAVHWFALGYDVVR.E
421	53 – 72	748.7458	2243.2155	2243.1375	34.8	0	55	0.0031	1	U	R.IPLEDGSAVHWFALGYDVVR.E
422	53 – 72	748.7477	2243.2214	2243.1375	37.4	0	66	0.00023	1	U	R.IPLEDGSAVHWFALGYDVVR.E
423	53 – 72	1122.6197	2243.2248	2243.1375	38.9	0	52	0.0057	1	U	R.IPLEDGSAVHWFALGYDVVR.E
424	53 – 72	748.7489	2243.2248	2243.1375	38.9	0	75	3.2e-005	1	U	R.IPLEDGSAVHWFALGYDVVR.E
81	65 – 72	446.7484	891.4823	891.4814	0.96	0	54	0.0089	1	U	F.ALGYDVVR.E
43	73 – 79	761.4032	760.3959	760.3967	-0.95	0	40	0.33	1	U	R.EVLGSEK.F
250	84 – 94	656.8112	1311.6078	1311.6030	3.64	0	45	0.047	1	U	R.VIGTHFNHQEM.A
439	84 – 110	766.9089	3063.6063	3063.5349	23.3	0	66	0.00015	1	U	R.VIGTHFNHQEMALPGNLLQLDPPEHTR.L
440	84 – 110	766.9097	3063.6097	3063.5349	24.4	0	49	0.0086	1	U	R.VIGTHFNHQEMALPGNLLQLDPPEHTR.L
441	84 – 110	766.9113	3063.6159	3063.5349	26.5	0	27	1.3	1	U	R.VIGTHFNHQEMALPGNLLQLDPPEHTR.L
442	84 – 110	1022.2127	3063.6164	3063.5349	26.6	0	22	4.3	1	U	R.VIGTHFNHQEMALPGNLLQLDPPEHTR.L
443	84 – 110	766.9132	3063.6236	3063.5349	29.0	0	53	0.0032	1	U	R.VIGTHFNHQEMALPGNLLQLDPPEHTR.L
444	84 – 110	766.9140	3063.6268	3063.5349	30.0	0	68	9.1e-005	1	U	R.VIGTHFNHQEMALPGNLLQLDPPEHTR.L
445	84 – 110	766.9141	3063.6273	3063.5349	30.2	0	61	0.00053	1	U	R.VIGTHFNHQEMALPGNLLQLDPPEHTR.L
446	84 – 110	766.9144	3063.6284	3063.5349	30.5	0	60	0.00057	1	U	R.VIGTHFNHQEMALPGNLLQLDPPEHTR.L
447	84 – 110	766.9149	3063.6303	3063.5349	31.2	0	48	0.0093	1	U	R.VIGTHFNHQEMALPGNLLQLDPPEHTR.L
448	84 – 110	766.9149	3063.6303	3063.5349	31.2	0	66	0.00017	1	U	R.VIGTHFNHQEMALPGNLLQLDPPEHTR.L
449	84 – 110	766.9154	3063.6325	3063.5349	31.9	0	62	0.00042	1	U	R.VIGTHFNHQEMALPGNLLQLDPPEHTR.L

Query	Start - End	Observed	Mr(expt)	Mr(calc)	ppm	M	Score	Expect	Rank	U	Peptide
450	84 - 110	1022.2184	3063.6335	3063.5349	32.2	0	72	4e-005	1	U	R.VIGTHFNHQEMALPGNLLQLDPPEHTR.L
451	84 - 110	766.9157	3063.6338	3063.5349	32.3	0	64	0.00022	1	U	R.VIGTHFNHQEMALPGNLLQLDPPEHTR.L
452	84 - 110	1022.2186	3063.6338	3063.5349	32.3	0	95	1.9e-007	1	U	R.VIGTHFNHQEMALPGNLLQLDPPEHTR.L
453	84 - 110	766.9159	3063.6346	3063.5349	32.6	0	54	0.0025	1	U	R.VIGTHFNHQEMALPGNLLQLDPPEHTR.L
454	84 - 110	766.9159	3063.6347	3063.5349	32.6	0	55	0.0019	1	U	R.VIGTHFNHQEMALPGNLLQLDPPEHTR.L
455	84 - 110	766.9160	3063.6350	3063.5349	32.7	0	66	0.00016	1	U	R.VIGTHFNHQEMALPGNLLQLDPPEHTR.L
456	84 - 110	613.7344	3063.6354	3063.5349	32.8	0	49	0.0073	1	U	R.VIGTHFNHQEMALPGNLLQLDPPEHTR.L
457	84 - 110	613.9356	3064.6415	3063.5349	361	0	74	2.2e-005	1	U	R.VIGTHFNHQEMALPGNLLQLDPPEHTR.L
346	95 - 110	885.9909	1769.9672	1769.9424	14.0	0	39	0.16	1	U	M.ALPGNLLQLDPPEHTR.L
251	100 - 110	659.8604	1317.7062	1317.7041	1.63	0	33	1	1	U	N.LLQLDPPEHTR.L
207	101 - 110	603.3274	1204.6402	1204.6200	16.7	0	15	59	1	U	L.LQLDPPEHTR.L
174	113 - 122	567.3174	1132.6203	1132.6175	2.46	1	25	5.8	1	U	R.RMVAPAFSVR.R
101	114 - 122	489.2715	976.5285	976.5164	12.4	0	54	0.009	1	U	R.MVAPAFSVR.R
102	114 - 122	489.2744	976.5342	976.5164	18.2	0	62	0.0013	1	U	R.MVAPAFSVR.R
112	123 - 130	500.7744	999.5342	999.5284	5.82	1	16	52	2	U	R.RMQALEPR.V
113	123 - 130	500.7749	999.5352	999.5284	6.88	1	32	1.4	1	U	R.RMQALEPR.V
57	124 - 130	422.7220	843.4295	843.4272	2.71	0	36	0.65	1	U	R.MQALEPR.V
58	124 - 130	422.7223	843.4300	843.4272	3.30	0	32	1.6	2	U	R.MQALEPR.V
59	124 - 130	422.7298	843.4451	843.4272	21.2	0	17	46	1	U	R.MQALEPR.V
429	131 - 153	837.7791	2510.3156	2510.2475	27.1	0	83	4.6e-006	1	U	R.VQAIVDDHLDTMASTGPPVEFLR.E
430	131 - 153	628.5890	2510.3268	2510.2475	31.6	0	46	0.024	1	U	R.VQAIVDDHLDTMASTGPPVEFLR.E
431	131 - 153	837.7835	2510.3285	2510.2475	32.3	0	67	0.00017	1	U	R.VQAIVDDHLDTMASTGPPVEFLR.E
432	131 - 153	837.7844	2510.3313	2510.2475	33.3	0	85	2.6e-006	1	U	R.VQAIVDDHLDTMASTGPPVEFLR.E
433	131 - 153	837.7848	2510.3326	2510.2475	33.9	0	96	2.5e-007	1	U	R.VQAIVDDHLDTMASTGPPVEFLR.E
434	131 - 153	628.5908	2510.3341	2510.2475	34.5	0	44	0.033	1	U	R.VQAIVDDHLDTMASTGPPVEFLR.E
64	147 - 153	429.2528	856.4911	856.4807	12.2	0	9	2.4e+002	1	U	G.PPVEFLR.E
65	147 - 153	429.2535	856.4925	856.4807	13.8	0	16	49	1	U	G.PPVEFLR.E
83	154 - 162	451.2318	900.4490	900.4487	0.37	0	41	0.19	1	U	R.EVAGPMAAR.V
84	154 - 162	451.2333	900.4521	900.4487	3.77	0	54	0.009	1	U	R.EVAGPMAAR.V
85	154 - 162	451.2335	900.4523	900.4487	4.03	0	54	0.009	1	U	R.EVAGPMAAR.V
86	154 - 162	451.2342	900.4539	900.4487	5.81	0	54	0.009	1	U	R.EVAGPMAAR.V
291	163 - 175	752.8991	1503.7837	1503.7392	29.6	0	64	0.00062	1	U	R.VACEFLGIPLDDR.G
292	163 - 175	752.9019	1503.7892	1503.7392	33.3	0	59	0.0023	1	U	R.VACEFLGIPLDDR.G
400	163 - 180	691.7170	2072.1293	2072.0724	27.4	1	49	0.012	1	U	R.VACEFLGIPLDDRGELIR.L
402	163 - 180	691.7259	2072.1560	2072.0724	40.3	1	110	1e-008	1	U	R.VACEFLGIPLDDRGELIR.L
165	192 - 201	560.7954	1119.5763	1119.5713	4.53	0	26	5.2	1	U	R.VLNGHAYLAY.M
265	192 - 203	704.3658	1406.7171	1406.7129	3.01	0	45	0.061	1	U	R.VLNGHAYLAYMR.E
267	192 - 203	470.2390	1407.6952	1406.7129	698	0	23	8.4	1	U	R.VLNGHAYLAYMR.E
268	192 - 203	704.8699	1407.7253	1406.7129	720	0	42	0.11	1	U	R.VLNGHAYLAYMR.E
269	192 - 203	470.2504	1407.7295	1406.7129	723	0	36	0.47	1	U	R.VLNGHAYLAYMR.E
209	212 - 223	609.7888	1217.5631	1217.5533	8.04	0	46	0.049	1	U	R.DPGDGMLGMVAR.D
210	212 - 223	609.7924	1217.5702	1217.5533	13.9	0	82	1.2e-005	1	U	R.DPGDGMLGMVAR.D
211	212 - 223	609.7953	1217.5760	1217.5533	18.7	0	67	0.0004	1	U	R.DPGDGMLGMVAR.D
212	212 - 223	609.7982	1217.5819	1217.5533	23.5	0	82	1.2e-005	1	U	R.DPGDGMLGMVAR.D
213	212 - 223	609.7983	1217.5821	1217.5533	23.6	0	12	1.3e+002	3	U	R.DPGDGMLGMVAR.D
214	212 - 223	609.7984	1217.5823	1217.5533	23.9	0	22	12	1	U	R.DPGDGMLGMVAR.D
215	212 - 223	609.7994	1217.5842	1217.5533	25.4	0	34	0.89	1	U	R.DPGDGMLGMVAR.D
216	212 - 223	609.7996	1217.5847	1217.5533	25.8	0	36	0.51	1	U	R.DPGDGMLGMVAR.D
217	212 - 223	609.8006	1217.5867	1217.5533	27.5	0	34	0.78	1	U	R.DPGDGMLGMVAR.D
218	212 - 223	609.8014	1217.5882	1217.5533	28.7	0	7	4e+002	2	U	R.DPGDGMLGMVAR.D
467	224 - 271	1042.7382	5208.6545	5208.5053	28.6	0	120	1.7e-010	1	U	R.DHGADISDEELAGLCAVVMNSSVEQTESCLAAGTLLLLEH
468	224 - 271	869.1167	5208.6565	5208.5053	29.0	0	169	2.3e-015	1	U	R.DHGADISDEELAGLCAVVMNSSVEQTESCLAAGTLLLLEH
469	224 - 271	869.1185	5208.6673	5208.5053	31.1	0	88	2.5e-007	1	U	R.DHGADISDEELAGLCAVVMNSSVEQTESCLAAGTLLLLEH
470	224 - 271	1042.7419	5208.6732	5208.5053	32.2	0	155	5.6e-014	1	U	R.DHGADISDEELAGLCAVVMNSSVEQTESCLAAGTLLLLEH
471	224 - 271	1042.7419	5208.6733	5208.5053	32.3	0	25	0.52	1	U	R.DHGADISDEELAGLCAVVMNSSVEQTESCLAAGTLLLLEH
464	243 - 271	1076.2506	3225.7299	3225.6339	29.8	0	113	2.6e-009	1	U	M.NSSVEQTESCLAAGTLLLLEHPEQFALLR.E
461	244 - 271	1038.2363	3111.6872	3111.5910	30.9	0	81	5.2e-006	1	U	N.SSVEQTESCLAAGTLLLLEHPEQFALLR.E
462	244 - 271	778.9300	3111.6907	3111.5910	32.0	0	72	4e-005	1	U	N.SSVEQTESCLAAGTLLLLEHPEQFALLR.E
322	272 - 286	585.3185	1752.9337	1752.9006	18.9	0	53	0.0074	1	U	R.ERPELGEQAVEEIVR.Y
323	272 - 286	585.3188	1752.9345	1752.9006	19.4	0	56	0.0031	1	U	R.ERPELGEQAVEEIVR.Y
324	272 - 286	585.3202	1752.9387	1752.9006	21.7	0	55	0.004	1	U	R.ERPELGEQAVEEIVR.Y
325	272 - 286	585.3202	1752.9387	1752.9006	21.7	0	60	0.0014	1	U	R.ERPELGEQAVEEIVR.Y
326	272 - 286	585.3236	1752.9491	1752.9006	27.7	0	58	0.0023	1	U	R.ERPELGEQAVEEIVR.Y
327	272 - 286	585.3240	1752.9502	1752.9006	28.3	0	18	23	1	U	R.ERPELGEQAVEEIVR.Y
330	272 - 286	585.3250	1752.9533	1752.9006	30.1	0	50	0.013	1	U	R.ERPELGEQAVEEIVR.Y
331	272 - 286	585.3253	1752.9542	1752.9006	30.6	0	54	0.0053	1	U	R.ERPELGEQAVEEIVR.Y
332	272 - 286	585.3255	1752.9546	1752.9006	30.8	0	51	0.011	1	U	R.ERPELGEQAVEEIVR.Y
333	272 - 286	585.3260	1752.9562	1752.9006	31.7	0	52	0.008	1	U	R.ERPELGEQAVEEIVR.Y
335	272 - 286	585.3264	1752.9573	1752.9006	32.3	0	52	0.0079	1	U	R.ERPELGEQAVEEIVR.Y
336	272 - 286	585.3264	1752.9574	1752.9006	32.4	0	57	0.0028	1	U	R.ERPELGEQAVEEIVR.Y
244	287 - 297	648.3546	1294.6947	1294.6557	30.1	0	63	0.00088	1	U	R.YLSVFEGLDPR.T
245	287 - 297	648.3549	1294.6952	1294.6557	30.4	0	57	0.0037	1	U	R.YLSVFEGLDPR.T
274	298 - 311	730.3967	1458.7788	1458.7566	15.3	0	94	6.1e-007	1	U	R.TATEDVEIGGQVIK.K
275	298 - 311	730.3982	1458.7818	1458.7566	17.3	0	96	4.4e-007	1	U	R.TATEDVEIGGQVIK.K
276	298 - 311	730.3986	1458.7826	1458.7566	17.8	0	98	2.9e-007	1	U	R.TATEDVEIGGQVIK.K
277	298 - 311	730.4006	1458.7866	1458.7566	20.6	0	89	2.2e-006	1	U	R.TATEDVEIGGQVIK.K
278	298 - 311	730.4020	1458.7895	1458.7566	22.5	0	89	2e-006	1	U	R.TATEDVEIGGQVIK.K
279	298 - 311	730.4045	1458.7944	1458.7566	26.0	0	75	4.9e-005	1	U	R.TATEDVEIGGQVIK.K
280	298 - 311	730.4045	1458.7945	1458.7566	26.0	0	82	1e-005	1	U	R.TATEDVEIGGQVIK.K
281	298 - 311	730.4047	1458.7949	1458.7566	26.2	0	62	0.00097	1	U	R.TATEDVEIGGQVIK.K
282	298 - 311	730.4050	1458.7954	1458.7566	26.6	0	71	0.00012	1	U	R.TATEDVEIGGQVIK.K
283	298 - 311	730.4053	1458.7960	1458.7566	27.0	0	68	0.00025	1	U	R.TATEDVEIGGQVIK.K
284	298 - 311	730.4058	1458.7971	1458.7566	27.8	0	79	1.9e-005	1	U	R.TATEDVEIGGQVIK.K
285	298 - 311	730.4060	1458.7974	1458.7566	28.0	0	84	6e-006	1	U	R.TATEDVEIGGQVIK.K
286	298 - 311	730.4064	1458.7983	1458.7566	28.6	0	72	0.00011	1	U	R.TATEDVEIGGQVIK.K
303	298 - 312	794.4405	1586.8665	1586.8515	9.42	1	47	0.029	1	U	R.TATEDVEIGGQVIKK.G
304	298 - 312	529.9628	1586.8665	1586.8515	9.43	1	56	0.0036	1	U	R.TATEDVEIGGQVIKK.G
295	312 - 325	768.4171	1534.8196	1534.7926	17.6	1	59	0.0022	1	U	K.KGEAVFCSLLAANR.A
296	312 - 325	768.4214	1534.8282	1534.7926	23.2	1	68	0.00024	1	U	K.KGEAVFCSLLAANR.A
266	313 - 325	704.3707	1406.7269	1406.6976	20.8	0	88	2.5e-006	1	U	K.GEAVFCSLLAANR.A
233	326 - 337	645.8335	1289.6524	1289.6252	21.1	0	77	3.8e-005	1	U	R.ADPALDGFDITR.K
234	326 - 337	645.8339	1289.6532	1289.6252	21.7	0	89	2.5e-006	1	U	R.ADPALDGFDITR.K
235	326 - 337	645.8359	1289.6573	1289.6252	24.9	0	78	3.4e-005	1	U	R.ADPALDGFDITR.K
236	326 - 337	645.8360	1289.6574	1289.6252	25.0	0	28	3.4	1	U	R.ADPALDGFDITR.K
237	326 - 337	645.8361	1289.6577	1289.6252	25.2	0	69	0.00023	1	U	R.ADPALDGFDITR.K

Query	Start - End	Observed	Mr(expt)	Mr(calc)	ppm	M	Score	Expect	Rank	U	Peptide
238	326 - 337	645.8363	1289.6580	1289.6252	25.5	0	40	0.2	1	U	R.ADPALDGFDITR.K
239	326 - 337	645.8384	1289.6622	1289.6252	28.7	0	26	4.9	1	U	R.ADPALDGFDITR.K
240	326 - 337	645.8389	1289.6633	1289.6252	29.5	0	34	0.77	1	U	R.ADPALDGFDITR.K
241	326 - 337	645.8391	1289.6636	1289.6252	29.8	0	36	0.5	1	U	R.ADPALDGFDITR.K
242	326 - 337	645.8402	1289.6659	1289.6252	31.6	0	34	0.81	1	U	R.ADPALDGFDITR.K
151	328 - 337	552.7987	1103.5828	1103.5611	19.6	0	43	0.1	1	U	D.PALDGFDITR.K
152	328 - 337	552.7988	1103.5831	1103.5611	19.9	0	25	7	2	U	D.PALDGFDITR.K
153	328 - 337	552.8000	1103.5854	1103.5611	22.0	0	25	7.8	1	U	D.PALDGFDITR.K
158	342 - 351	556.2845	1110.5544	1110.5472	6.52	0	25	5.5	1	U	R.HVAFGHGIHH.C
230	342 - 352	636.2962	1270.5777	1270.5778	-0.057	0	41	0.13	1	U	R.HVAFGHGIHHC.L
317	342 - 357	574.9660	1721.8762	1721.8573	11.0	0	18	23	1	U	R.HVAFGHGIHHCLGAPL.A
370	342 - 359	650.6851	1949.0335	1948.9955	19.5	0	52	0.0078	1	U	R.HVAFGHGIHHCLGAPLAR.M
371	342 - 359	488.2657	1949.0335	1948.9955	19.5	0	47	0.025	1	U	R.HVAFGHGIHHCLGAPLAR.M
372	342 - 359	650.6881	1949.0425	1948.9955	24.1	0	21	8.8	1	U	R.HVAFGHGIHHCLGAPLAR.M
374	342 - 359	650.6908	1949.0505	1948.9955	28.2	0	8	1.9e+002	2	U	R.HVAFGHGIHHCLGAPLAR.M
375	342 - 359	650.6913	1949.0520	1948.9955	29.0	0	14	53	1	U	R.HVAFGHGIHHCLGAPLAR.M
376	342 - 359	488.2706	1949.0535	1948.9955	29.7	0	3	5.6e+002	8	U	R.HVAFGHGIHHCLGAPLAR.M
377	342 - 359	488.2711	1949.0555	1948.9955	30.8	0	4	5.3e+002	10	U	R.HVAFGHGIHHCLGAPLAR.M
378	342 - 359	650.6925	1949.0556	1948.9955	30.9	0	17	24	1	U	R.HVAFGHGIHHCLGAPLAR.M
379	342 - 359	488.2713	1949.0560	1948.9955	31.0	0	5	3.9e+002	9	U	R.HVAFGHGIHHCLGAPLAR.M
380	342 - 359	650.6928	1949.0565	1948.9955	31.3	0	11	1.1e+002	1	U	R.HVAFGHGIHHCLGAPLAR.M
381	342 - 359	650.6930	1949.0573	1948.9955	31.7	0	11	87	1	U	R.HVAFGHGIHHCLGAPLAR.M
382	342 - 359	650.6931	1949.0574	1948.9955	31.7	0	21	8.7	1	U	R.HVAFGHGIHHCLGAPLAR.M
383	342 - 359	488.2717	1949.0577	1948.9955	31.9	0	9	1.6e+002	2	U	R.HVAFGHGIHHCLGAPLAR.M
384	342 - 359	650.6934	1949.0585	1948.9955	32.3	0	12	77	1	U	R.HVAFGHGIHHCLGAPLAR.M
385	342 - 359	488.2721	1949.0592	1948.9955	32.7	0	7	2.4e+002	4	U	R.HVAFGHGIHHCLGAPLAR.M
386	342 - 359	488.2722	1949.0595	1948.9955	32.8	0	9	1.5e+002	4	U	R.HVAFGHGIHHCLGAPLAR.M
387	342 - 359	650.6941	1949.0605	1948.9955	33.4	0	7	2.4e+002	2	U	R.HVAFGHGIHHCLGAPLAR.M
389	342 - 359	488.2725	1949.0610	1948.9955	33.6	0	7	2.3e+002	5	U	R.HVAFGHGIHHCLGAPLAR.M
289	346 - 359	499.2660	1494.7762	1494.7626	9.10	0	29	2	1	U	F.GHGIHHCLGAPLAR.M
290	346 - 359	748.3959	1494.7773	1494.7626	9.80	0	45	0.047	1	U	F.GHGIHHCLGAPLAR.M
247	348 - 359	651.3500	1300.6853	1300.6823	2.38	0	29	2.3	1	U	H.GIHHCLGAPLAR.M
117	364 - 372	504.3014	1006.5883	1006.5811	7.15	0	66	0.00042	1	U	R.IAFTTLVSR.F
118	364 - 372	504.3040	1006.5935	1006.5811	12.3	0	66	0.00039	1	U	R.IAFTTLVSR.F
119	364 - 372	504.3101	1006.6056	1006.5811	24.3	0	66	0.0003	1	U	R.IAFTTLVSR.F
120	364 - 372	504.3110	1006.6075	1006.5811	26.2	0	56	0.0031	1	U	R.IAFTTLVSR.F
121	364 - 372	504.3112	1006.6078	1006.5811	26.5	0	47	0.024	2	U	R.IAFTTLVSR.F
122	364 - 372	504.3112	1006.6079	1006.5811	26.6	0	65	0.00039	1	U	R.IAFTTLVSR.F
123	364 - 372	504.3113	1006.6080	1006.5811	26.7	0	52	0.0084	1	U	R.IAFTTLVSR.F
124	364 - 372	504.3113	1006.6081	1006.5811	26.8	0	65	0.00038	1	U	R.IAFTTLVSR.F
125	364 - 372	504.3116	1006.6086	1006.5811	27.3	0	66	0.00032	1	U	R.IAFTTLVSR.F
105	378 - 386	493.2741	984.5336	984.5240	9.75	0	54	0.0072	1	U	R.TAVPAEEIR.F
106	378 - 386	493.2746	984.5347	984.5240	10.9	0	54	0.0072	1	U	R.TAVPAEEIR.F
140	387 - 395	525.7761	1049.5375	1049.5294	7.73	0	26	5.6	1	U	R.FRPPSSNVF.T
141	387 - 395	525.7792	1049.5438	1049.5294	13.7	0	23	9.7	1	U	R.FRPPSSNVF.T
405	387 - 404	1059.1043	2116.1940	2116.1357	27.5	0	48	0.015	1	U	R.FRPPSSNVFTLLELPLTW.-
406	387 - 404	1059.1077	2116.2008	2116.1357	30.8	0	79	1.1e-005	1	U	R.FRPPSSNVFTLLELPLTW.-
407	387 - 404	706.4103	2116.2090	2116.1357	34.6	0	65	0.00026	1	U	R.FRPPSSNVFTLLELPLTW.-
408	387 - 404	1059.1118	2116.2090	2116.1357	34.6	0	61	0.00077	1	U	R.FRPPSSNVFTLLELPLTW.-

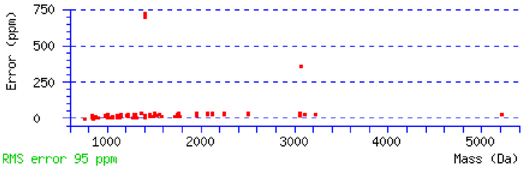

Figure S4. Verification of OxyA$_{kis}$ Y99F mutation by peptide fingerprinting.

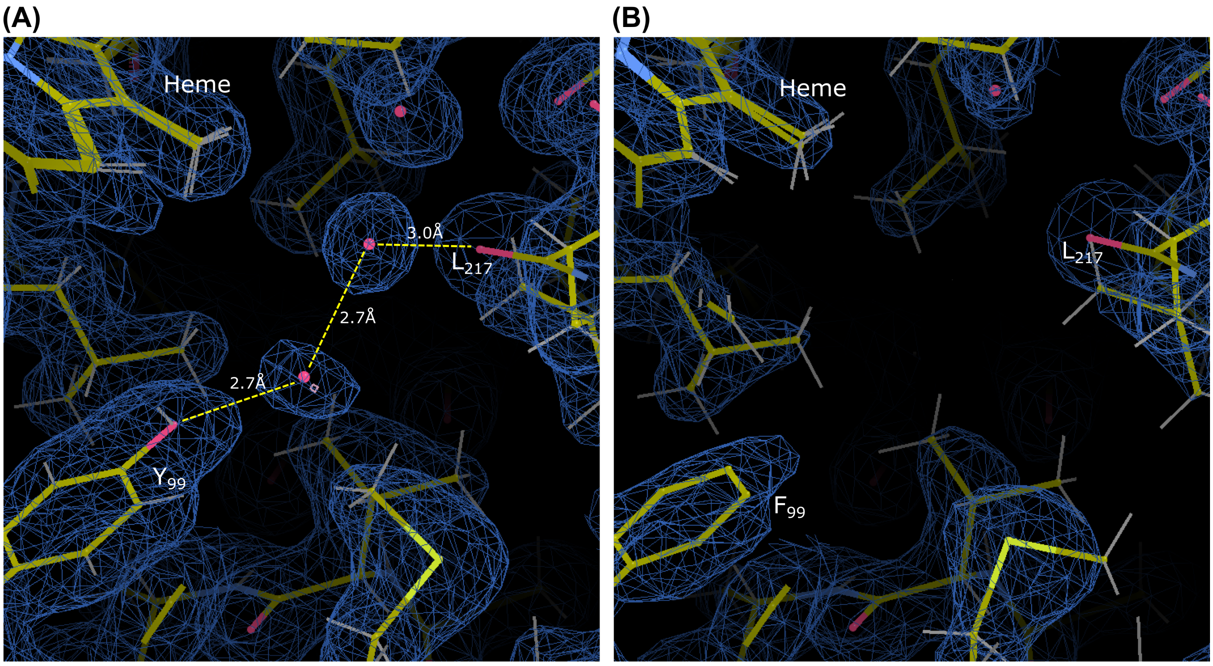

Figure S5. Comparison of electron density of Y99 and mutated F99 residue in OxyA$_{kis}$ structures. (A) Wildtype OxyA$_{kis}$ with water mediated hydrogen bonding network between Tyr99 and Leu217. Hydrogen bonds shown as dashed yellow lines (distances as indicated). (B) OxyA$_{kis}$ Tyr99Phe mutant structure showing a lack of electron density for these water molecules. Electron density map shown as blue mesh (1.0σ 2mFo-DFc map). Water molecules shown as red spheres; carbons atoms: tan – oxygen atoms – red; nitrogen atoms – blue; sulfur atoms – yellow. Figures generated in COOT.[2]

SI References.

1. Sievers, F.; Wilm, A.; Dineen, D.; Gibson, T. J.; Karplus, K.; Li, W.; Lopez, R.; McWilliam, H.; Remmert, M.; Söding, J.; Thompson, J. D.; Higgins, D. G., Fast, scalable generation of high-quality protein multiple sequence alignments using Clustal Omega. *Mol Sys Biol* **2011,** *7* (1).
2. Emsley, P.; Cowtan, K., Coot: model-building tools for molecular graphics. *Acta Crystallogr D Biol Crystallogr* **2004,** *60* (Pt 12 Pt 1), 2126-32.
